# Noninvasive microbiota-based diagnostic models for pancreatic ductal adenocarcinoma: a systematic review and diagnostic test accuracy meta-analysis

**DOI:** 10.3389/fmicb.2026.1866794

**Published:** 2026-09-23

**Authors:** Liang Zhang, Jian Liu, Mengyao Pang, Chenfei Liang, Da Li, Yali Wang

**Affiliations:** Department of Gastroenterology, Beijing Huaxin Hospital, The First Hospital of Tsinghua University, Beijing, China

**Keywords:** diagnostic test accuracy, meta-analysis, microbiota, pancreatic ductal adenocarcinoma, saliva, stool, systematic review, tongue coat

## Abstract

**Background:**

Due to delays in diagnosis, many patients with pancreatic ductal adenocarcinoma (PDAC) miss the window for curative interventions. The oral and gut microbiomes are attractive as noninvasive triage instruments; however, while the literature provides AUROC values, it has not established clinically useful thresholds.

**Objective:**

To systematically assess the diagnostic performance and clinical maturity of noninvasive microbiota-derived tools for identifying PDAC.

**Methods:**

Following PRISMA 2020 and PRISMA-DTA, we searched eight databases up to April 19, 2026, and conducted a targeted supplementary search on September 3, 2026. Risk of bias was assessed using PROBAST and QUADAS-2. The nine-row meta-analysis, which included approximations and correlated estimates, was exploratory.

**Results:**

The initial search and targeted supplementary search yielded 16 studies covering 21 sample-domain items; 20 model-based records constituted the units of analysis for the risk-of-bias assessment. Fifty-one AUROC estimates formed a separate list of discrimination estimates. Six-row fixed-threshold data from five studies constituted the primary descriptive core. Sensitivity ranged from 0.682 to 0.964, and specificity ranged from 0.667 to 0.963. The exploratory nine-row bivariate model yielded pooled sensitivity of 0.86 (95% CI 0.80–0.90) and specificity of 0.80 (95% CI 0.73–0.86). These nine rows represented seven studies; Li 2025 and Chen 2025b each provided a correlated row, so the pooled estimates were hypothesis-generating. The certainty of evidence was rated very low using an adapted, GRADE-informed appraisal. All model-based rows (20/20) were at high risk of bias in the PROBAST analysis domain and high overall risk on QUADAS-2. Although stool-based testing provided the clearest signal of external validation, benign pancreatic mimics remained underrepresented.

**Conclusion:**

Detecting PDAC via noninvasive microbiota classifiers carries a biologically plausible yet very low-certainty diagnostic signal. Current findings do not support stand-alone clinical deployment and support only exploratory adjunctive triage. Validating prospectively against benign pancreatic mimics is mandatory before routine clinical adoption.

**Systematic review registration:**

https://www.crd.york.ac.uk/PROSPERO/view/CRD420261370926, Identifier, CRD420261370926.

## Introduction

Because the window of opportunity for curative resection is typically quite limited, pancreatic ductal adenocarcinoma continues to have an extremely high mortality rate among cancers of the digestive system. Early disease presents silently; thus, most patients are recognized far too late for surgical intervention. Current diagnostic pathways rely on imaging and tissue sampling, with CA19-9 used as an adjunctive biomarker (Ballehaninna and Chamberlain, 2012).

In routine care, CA19-9 is still the most frequently used biomarker for pancreatic cancer, but its utility depends entirely on context. In practice, it supports diagnostic efforts while providing essential metrics for both response monitoring and postoperative surveillance. Yet, as a stand-alone screening tool—particularly in low-prevalence environments—it is not sufficiently specific or sensitive. At the conventional 37 U/mL threshold, CA19-9 has 79–81% sensitivity and 82–90% specificity in symptomatic patients, but its low positive predictive value limits population screening (Ballehaninna and Chamberlain, 2012).

Saliva and stool samples are easy to collect, which lends microbial profiling techniques both practical value and biological plausibility. Given the continuous interaction between the pancreaticobiliary tract, the gut, and the oral cavity, they are not biologically discrete. This systemic interconnectivity has a clear effect. PDAC is associated with microbiota changes that we can measure noninvasively. These changes can be observed in saliva, stool, tongue coating, and oral rinse samples. However, achieving high diagnostic discriminatory power on these platforms has involved a stepwise evolution: an early milestone was the oral assessment by Farrell et al. (2012). Researchers in this field eventually redirected their focus toward complex stool-based diagnostic testing. Early modeling by Ren et al. (2017) laid the groundwork, followed by successive publications from Kartal et al. (2022), Nagata et al. (2022), and Sammallahti et al. (2025) demonstrating externally validated stool microbiota classifiers. Building upon these results, subsequent reports have investigated microbial features across multiple sample types, including stool, the oral cavity, and tongue coating (Half et al., 2019; Lu et al., 2019; Zhou et al., 2021; Chen et al., 2023; Yang et al., 2023; Li et al., 2024; Chen Y. et al., 2025; Li et al., 2025; Tavanaeian et al., 2025; Villani et al., 2025; Fujii et al., 2026).

How to validate these methods based on core criteria in relevant patient populations and achieve precise diagnosis of PDAC using specific microbiota score thresholds remains a major technical challenge.

Therefore, this paper categorizes the existing literature based on evidence maturity. All eligible noninvasive microbiota studies related to the diagnosis of PDAC fall into a broader review layer. Meanwhile, the quantitative layer is segmented: it separates a higher-confidence, fixed-threshold DTA core from both an expanded exploratory sensitivity set and a completely distinct AUROC-only evidence layer. Because this structured framework rests on clinical interpretability, it helps prevent the field from overstating the maturity of discrimination-only cohorts (Farrell et al., 2012; Ren et al., 2017; Half et al., 2019; Lu et al., 2019; Zhou et al., 2021; Kartal et al., 2022; Nagata et al., 2022; Chen et al., 2023; Yang et al., 2023; Li et al., 2024; Chen Y. et al., 2025; Li et al., 2025; Sammallahti et al., 2025; Tavanaeian et al., 2025; Villani et al., 2025; Fujii et al., 2026).

## Methods

### Study design and registration

The PRISMA 2020 and PRISMA-DTA statements (McInnes et al., 2018; Page et al., 2021) served as the methodological basis for conducting this systematic review, as well as the accompanying diagnostic test accuracy synthesis. The protocol was registered with PROSPERO on April 16, 2026, under registration number CRD420261370926. Although we had already begun preliminary literature searches and screening, this registration was completed before the finalization of the study inclusion criteria and the conduct of any quantitative synthesis.

### Eligibility criteria

Throughout this review, “screening” refers to testing of asymptomatic individuals; “early detection” targets potentially curable disease; “surveillance” refers to serial assessment of high-risk individuals; “diagnosis” refers to evaluations prompted by symptoms or abnormal findings; and “triage” refers to auxiliary tests used to determine the priority of imaging studies, endoscopic ultrasound (EUS), or tissue confirmation. To qualify for our analysis, adult studies needed to focus on PDAC or allied pancreatic cancer subtypes, as long as data for a predominant PDAC majority could be explicitly isolated. Noninvasive microbial sources—stool, oral samples, mouth rinses, and tongue coatings—were the only index tests evaluated. In our core analysis, we specifically excluded invasive sampling techniques such as biliary sampling, tissue biopsy, and ductal brushing. To be eligible for inclusion, studies had to report usable diagnostic metrics (e.g., AUC, sensitivity, specificity, or ROC curves). Although descriptive microbiome reports lacking these metrics might contribute to biological interpretation, they did not meet the criteria of our DTA analysis.

The specific disease focus for this analysis was PDAC. Investigations employing either multi-disciplinary center adjudication or hybrid clinico-pathological diagnostic routes were deemed eligible for our review, even though cytologic and histopathologic verification remained the overwhelmingly preferred diagnostic reference standard. We captured these differences because they are pivotal to assessing bias and the applicability of the findings. Although healthy control groups are frequently reported in the literature, we determined that low-grade pancreatic lesions, chronic pancreatitis, and benign conditions are substantially more clinically relevant for the purposes of our diagnostic review.

### Search strategy and review layers

We searched the Cochrane Library, Web of Science, Scopus, Embase, PubMed, VIP, Wanfang, and CNKI. The search terms combined concepts such as PDAC, microbiome, non-invasive sampling, sequencing, and diagnostic performance; see Supplementary Table S3 and the audit workbook for details on the specific search strategies used in each database and the number of records retrieved. The initial database search was completed on April 19, 2026. On September 3, 2026, we conducted a targeted supplementary search of PubMed and Europe PMC to capture records entered between April 20 and September 3, 2026. This supplementary search identified 35 records (16 from PubMed; 19 from Europe PMC); after excluding 15 duplicate records across databases and 1 record already present in the original search results, 19 records underwent title/abstract screening, of which 3 were reviewed in full text, ultimately resulting in the inclusion of 1 newly eligible report. A subsequent expanded verification did not identify any other eligible reports. We deliberately separated the broad systematic review level from the targeted DTA synthesis to avoid confusion between the two sets of inclusion criteria. A locked data extraction repository, consisting solely of studies with usable diagnostic metrics, supported the quantitative synthesis. Figure 1 illustrates the study screening process.

**Figure 1:**
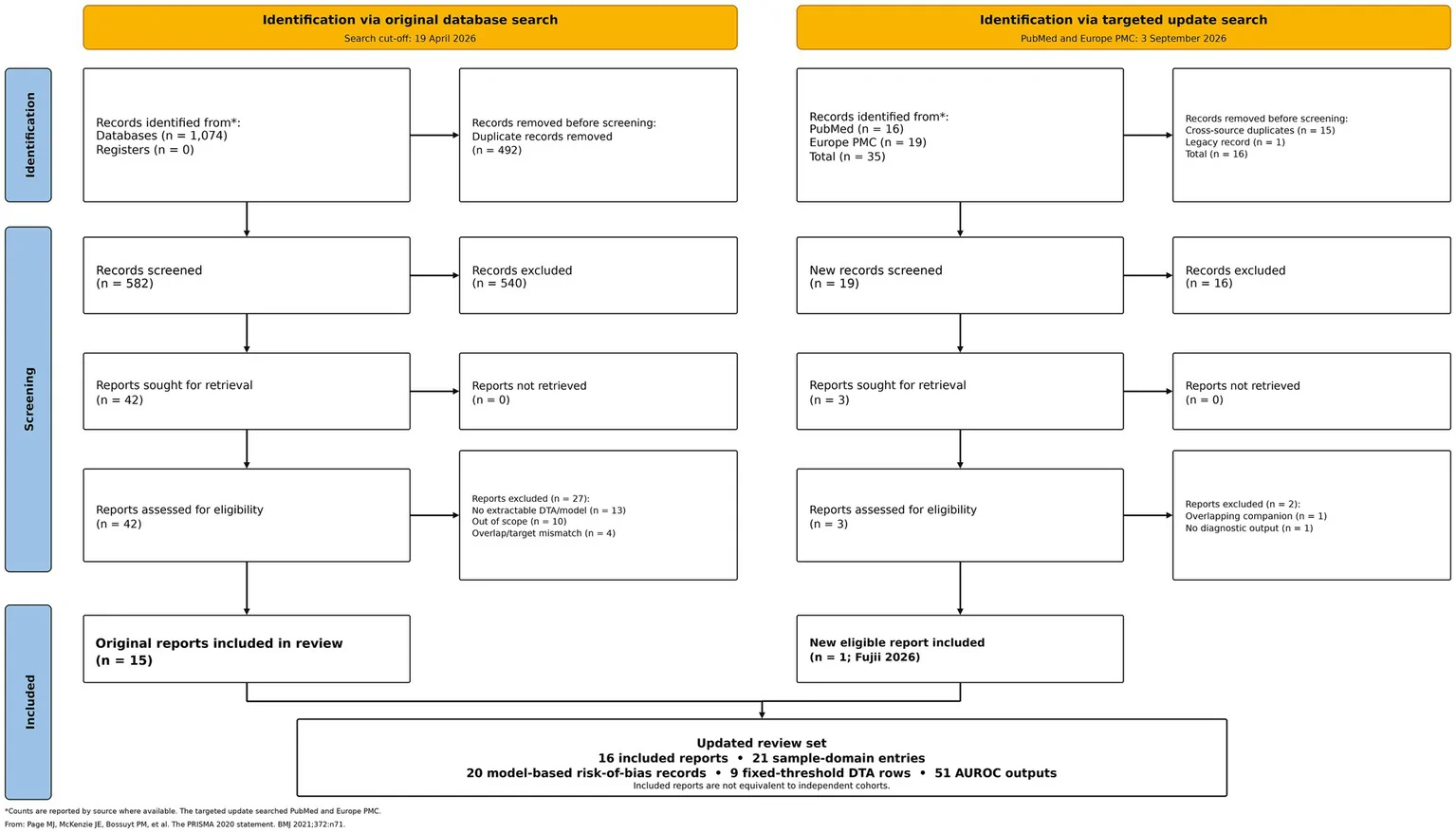
PRISMA 2020 flow diagram. The original eight-database search identified 1,074 records through April 19, 2026. The *post-hoc* targeted PubMed/Europe PMC top-up through September 3, 2026 identified 35 records, removed 16 duplicate or previously screened records, screened 19, assessed three in detail, and included one newly eligible report. The updated review contains 16 reports/publications, 21 sample-domain entries, 20 model-based risk-of-bias records, and 51 descriptive AUROC estimates. The top-up was not a rerun of all eight databases; report counts are not independent-cohort counts. Adapted from the PRISMA 2020 flow-diagram template (Page et al., 2021), licensed under CC BY 4.0.

### Data extraction and evidence structuring

Data extraction was based on a unified database. This architecture mapped 2 × 2 tables with fixed thresholds and microbiome reporting compliance to the characteristics of each study. Supplementary Tables S4–S6 contain the AUROC models, reconstruction logs, and audit sets. We have documented all data extraction methods and analysis procedures, preserving strict auditability over any technical heterogeneity. Evidence was organized into four review layers: broad review, analysis database, fixed-threshold DTA, and AUROC-only. Separately, A/B/C denoted review-specific data-reconstruction verification tiers developed during data extraction and fixed before the final quantitative synthesis; they were not an established DTA evidence-grading system and did not replace QUADAS-2, PROBAST, or the certainty appraisal. Tier A required directly reported or exactly recoverable integer 2 × 2 cells; Tier B required near-integer recovery from a clearly defined validation sample with consistency checks against reported performance measures; and Tier C comprised approximate reconstructions used only in exploratory sensitivity analyses. The “Exploratory” label in Table 1 denotes eligible descriptive entries with reported diagnostic performance but unstable or internally selected thresholds that did not meet A–C reconstruction criteria; these entries were not pooled. Updated counts included 16 studies and 21 sample-domain analyses (20 acting as the risk-of-bias unit). We logged nine fixed-threshold rows, six Tier A/B primary rows, and 51 AUROC estimates. Li 2025 and Chen 2025b each contributed paired overlapping fixed-threshold rows, requiring explicit handling in the dependence sensitivity analyses.

**Table 1:** Sample-domain characteristics of included noninvasive microbiota studies for PDAC.

Study/entry	Country	Sample	Review layer/verification tier	Control type	Method	Reported values
Farrell 2012	USA	Oral/saliva	Core / Tier A	Healthy	microarray/qPCR	AUC 0.900; Se 0.964; Sp 0.821
Nagata 2022 [oral]	JP/ES/DE	Oral/saliva	AUROC	Mixed	shotgun	AUC 0.820
Chen 2023 [oral]	China	Oral/saliva	AUROC	Healthy	16S	AUC 0.916
Li 2024 [oral]	China	Oral/saliva	AUROC	Benign pancreatic	16S	AUC 0.963
Li 2025 [oral]	China	Oral/saliva	Core / Tier B	Healthy	16S	AUC 0.963; Se 0.930; Sp 0.970
Tavanaeian 2025	Iran	Oral/saliva	Exploratory	Healthy	qPCR/16S	AUC 0.970
Ren 2017	China	Stool	Core / Tier A	Healthy	16S	AUC 0.842; Se 0.859; Sp 0.667
Half 2019	Israel	Stool	Exploratory	Mixed	16S	AUC 0.825; Se 0.770; Sp 0.800
Zhou 2021	China	Stool	AUROC	Healthy; AIP 2nd	shotgun	AUC 0.907
Kartal 2022	ES/DE	Stool	AUROC	Mixed non-pancreatic	shotgun/16S	AUC 0.840
Nagata 2022 [stool]	JP/ES/DE	Stool	AUROC	Mixed	shotgun	AUC 0.780
Chen 2023 [stool]	China	Stool	AUROC	Healthy	16S	AUC 0.856
Yang 2023	China	Stool	AUROC	Healthy	16S	AUC 0.927
Li 2024 [stool]	China	Stool	AUROC	Benign pancreatic	16S	AUC 0.894
Chen 2025b†	China	Stool	Expanded / Tier C	Healthy	shotgun	AUC 0.923; Se 0.840; Sp 0.840
Li 2025 [stool]	China	Stool	Core / Tier B	Healthy	16S	AUC 0.890; Se 0.880; Sp 0.750
Villani 2025	Italy	Stool	Exploratory	Healthy	16S	AUC 1.000; Se/Sp unstable
Lu 2019	China	Tongue coat	Expanded / Tier C	Healthy	16S	AUC 0.802; Se 0.771; Sp 0.786
Sammallahti 2025	FI/IR	Stool	Core / Tier A; external	Healthy	16S	AUC 0.880; Se 0.850; Sp 0.890
Tavanaeian 2025 [stool]	Iran	Stool	AUROC	Healthy	qPCR/16S	AUC 1.000; Se/Sp inconsistent
Fujii 2026	Japan	Stool	Core / Tier A	Healthy; high-risk secondary	targeted qPCR	AUC 0.780; Se 0.682; Sp 0.723

Reported performance values are retained as originally reported; reconstructed 2 × 2-derived values used for DTA analyses are shown separately in Table 2. All DTA analyses are based exclusively on the reconstructed values in Table 2; this table is descriptive only. † Chen 2025b is one study-sample entry contributing two overlapping fixed-threshold model rows (M1 and M2) to Table 2 and the k = 9 exploratory analyses. Fujii 2026 was identified in the targeted top-up and contributes one Tier A fixed-threshold row.

Information retrieved during extraction spanned the country of study and sample site to the sequencing platform used, incorporating vital data on comparator types, study layouts, and validation procedures. Total counts for cases and controls, sensitivity, specificity, AUROC values, and the source of the thresholds were also recorded, along with the status of 2 × 2 table reconstruction. We employed a hierarchical validation approach (including external validation, internal hold-out validation, resampling, or whole-cohort validation) to avoid overestimating model performance due to internal optimization for accuracy. To reflect the clinical differences between the PDAC group and the healthy control group, as well as between the PDAC group and the benign disease control group, the comparator structure in this study was recorded as a standalone variable.

Given that microbiome data are susceptible to pre- and post-analytic biases, we extracted information on treatment-naive status, antibiotic/PPI exposure, sequencing region, contamination and quality control, batch correction, and validation maturity. Because data completeness and the number of studies were insufficient to perform a meta-regression, the above variables were included in a structured narrative review (to assess generalizability) but were not modeled as effect modifiers.

### 2 × 2 reconstruction rules

Reconstructing 2 × 2 tables was a precise process, and it required a strict structure. Studies that directly provided raw values for true positives, false positives, true negatives, and false negatives were given the highest priority. As an alternative, a secondary approach allowed us to combine the total number of cases with sensitivity and specificity values at a fixed threshold to reconstruct the four-cell integer data. Entries lacking consistent thresholds or necessary data were assigned directly to the AUROC-only review layer. Each row was then classified into the verification tiers defined above for this review. Each row of reconstructed data was accompanied by a source label. The relevant information was obtained through direct reporting, digitization, estimation, inference based on sample size and accuracy of the validation split, or mathematical derivation. Continuity adjustments and rounding rules were tracked in our reconstruction log. Wilson intervals calculated from reconstructed cell counts quantify binomial sampling uncertainty; however, they do not account for supplemental uncertainty caused by data rounding, digitization, validation-split inferences, or model selection. Consequently, Tier C rows were kept out of the primary core and reserved for exploratory analyses.

### Statistical analysis

Rather than pooling the specificity and sensitivity within the primary core (six rows from five studies), we reported the metrics for each study along with their 95% Wilson confidence intervals. To determine whether any single row dominated the descriptive pattern, we conducted a row-level leave-one-out analysis; this check did not model dependence between the paired Li 2025 oral and stool rows. Therefore, for this six-row core, we did not calculate pooled estimates of sensitivity or specificity.

We fitted a bivariate random-effects model to the 9 rows of data from the 7 studies (maintaining an exploratory approach due to the inclusion of Tier C reconstructions and correlated rows). Due to the lack of individual covariances, treating correlated rows as independent may have underestimated statistical uncertainty and imbalanced weight allocation. Therefore, the analysis excluding Chen’s two rows was retained, and a *post hoc* deterministic one-row-per-study sensitivity analysis was added: data were screened according to predefined rules (in the order of validation maturity > verification tier > order of appearance in the source report, independent of observed diagnostic performance). τ^2^ and I^2^ from marginal univariate models were used to describe heterogeneity; given the small number of independent studies, caution was exercised in interpreting the prediction intervals.

Using an adapted, GRADE-informed framework, the certainty of the fixed-threshold diagnostic evidence was assessed in terms of risk of bias, indirectness, inconsistency, imprecision, and publication bias (Schünemann et al., 2020a, 2020b). The assessment of risk of bias was based on the QUADAS-2 and PROBAST criteria; the assessment of indirectness was based on the relevance of the comparator and the target setting; the assessment of inconsistency was based on between-study variability; and the assessment of imprecision was based on the width of confidence intervals and the number of independent studies. The specific rationale for each domain is shown in Figure 2. This appraisal was not a formally validated GRADE-DTA tool; we used PRISMA-DTA item 24 solely for reporting structure.

**Figure 2:**
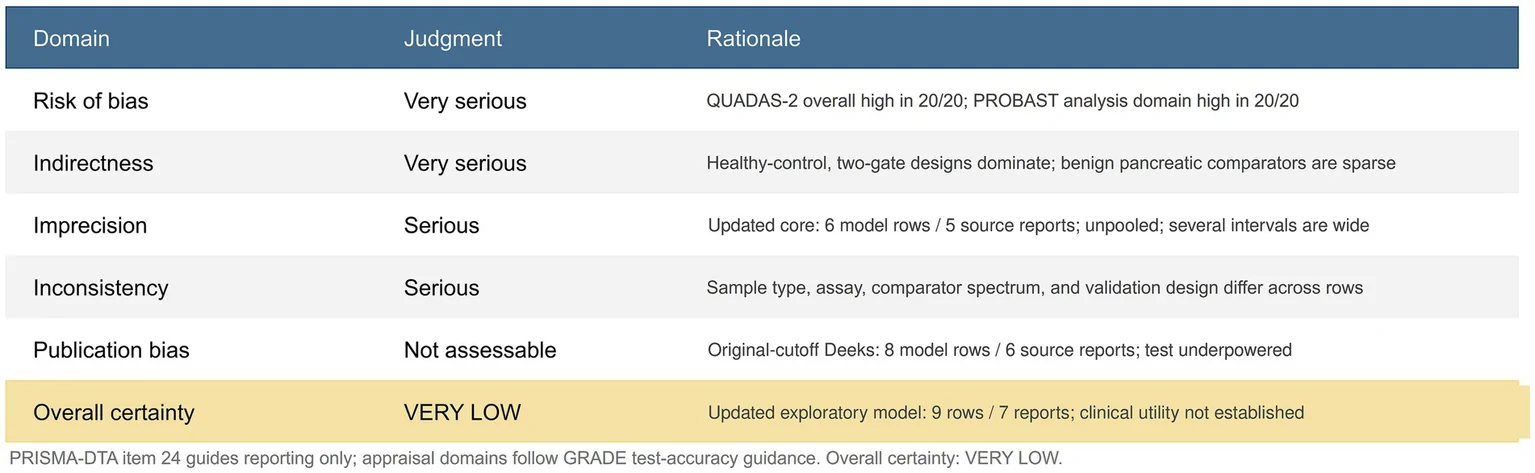
Adapted, GRADE-informed certainty appraisal. Risk of bias, indirectness, inconsistency, imprecision, and publication bias were appraised with explicit rationales (Schünemann et al., 2020a, 2020b); overall certainty was very low. The updated primary descriptive core contains six model rows from five source reports and remains unpooled. The nine-row/seven-report synthesis is exploratory; the eight-row/six-report original-cutoff Deeks test is underpowered. PRISMA-DTA item 24 guides reporting, not appraisal.

Due to the limited number of studies (k < 10), the Deeks test (Deeks et al., 2005) was prespecified solely for exploratory assessment of small-sample study effects and was not used to assert the absence of publication bias. The analytical software used included R 4.3.3 (GLMMadaptive, mada) and Python 3.12.13 (NumPy and SciPy). GLMMadaptive’s same-binomial-likelihood check agreed with Python for sensitivity, specificity, and four 95% confidence limits to three decimals; we used the mada package in R to perform a normal–normal method-sensitivity check of the output from the Python workflow. Stata was not run (Supplementary Methods M2).

## Results

### Study selection and evidence layers

Through an initial search and targeted supplementary searches (35 records identified; 16 duplicate or previously screened records removed; 19 screened; three assessed in detail; one newly eligible report included), 16 studies and 21 sample-domain analysis entries were ultimately included. Eight study-sample entries yielded nine final fixed-threshold model rows with reconstructable 2 × 2 tables: three Tier C rows and six Tier A/B rows. These entries moved directly into the quantitative DTA layer. An inability to plot one entry as a model-based risk-of-bias row separates the 21 analysis entries from the 20 risk-of-bias rows. Because they lacked stable fixed-threshold 2 × 2 information, 13 entries remained confined to the non-final-DTA descriptive layers, including 10 AUROC-only entries and 3 exploratory candidates. We constructed the primary descriptive DTA core using six Tier A/B fixed-threshold rows from five studies. The nine-row bivariate model functioned exclusively as an exploratory sensitivity analysis. For estimates based solely on AUROC, we presented them in the form of descriptive charts. Fujii et al. (2026) identified in the targeted top-up, contributed one Tier A stool row and three AUROC estimates. The expanded nine-row synthesis represented seven studies; Li 2025 and Chen 2025b each contributed correlated rows. Removing both Chen 2025b rows left seven rows from six studies and yielded bivariate sensitivity 0.86 (95% CI 0.80–0.91) and specificity 0.80 (95% CI 0.71–0.87); corresponding univariate marginal estimates were 0.84 (95% CI 0.76–0.89) and 0.77 (95% CI 0.69–0.84). A *post-hoc* deterministic one-row-per-study analysis across seven studies yielded bivariate sensitivity 0.86 (95% CI 0.79–0.91) and specificity 0.82 (95% CI 0.72–0.88). These sensitivity analyses were directionally consistent but remained hypothesis-generating.

Table 1 summarizes the study characteristics for all 21 sample-domain analysis entries. To facilitate direct review, this model-driven table summarizes the sample domains, comparator structure, sequencing platforms, reported diagnostic performance, and assigned review layer and verification tier.

### Risk-of-bias assessment

Frequent concerns arose throughout PROBAST (Wolff et al., 2019) and QUADAS-2 (Whiting et al., 2011) assessments. Persistent flaws in study design systematically compromised index-test procedures, patient recruitment, clinical applicability, and the final model evaluation outcomes. The limited external validation and prevalent case–control enrichment of the present evidence base supported these judgments. Reviewing the 20 model-based risk-of-bias rows (Figure 3), QUADAS-2 scored patient selection as high risk in 16/20 instances (80%). Every overall QUADAS-2 judgment was high risk (20/20 instances, 100%). PROBAST identified equally critical flaws. High risk categorized the analysis domain in 20/20 evaluations (100%) and the overall PROBAST judgment in 20/20 evaluations (100%). Optimistic discrimination, case–control enrichment, and internal-only validation principally drove these figures.

**Figure 3:**
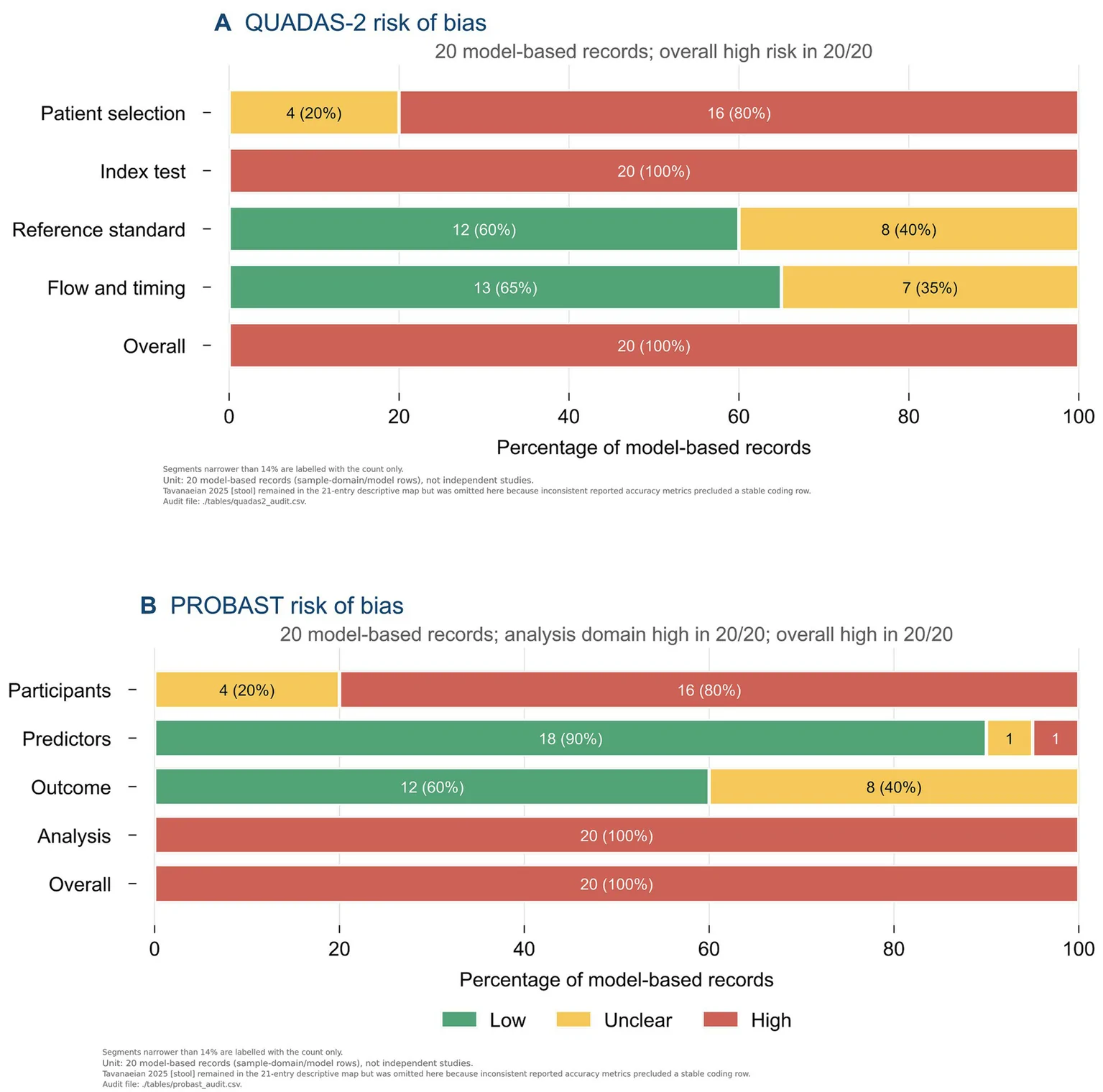
Risk-of-bias summaries. Panel **A** shows QUADAS-2 judgments and Panel **B** shows PROBAST judgments across 20 model-based records. All overall QUADAS-2 judgments and all PROBAST analysis-domain judgments were high risk.

The adapted, GRADE-informed appraisal rated risk of bias very serious, indirectness serious to very serious, imprecision serious, inconsistency serious, and publication bias not assessable, resulting in an overall certainty of evidence rated very low (Figure 2; Schünemann et al., 2020a, 2020b). This assessment is reported under PRISMA-DTA item 24; that item is a reporting standard, not an appraisal method.

### Primary fixed-threshold DTA core and expanded sensitivity analysis

The central pillar of our primary interpretation relied on six fixed-threshold rows meeting verification Tier A or B criteria. Farrell 2012, Ren 2017, Sammallahti 2025, Fujii 2026, and Li 2025 (stool and oral evaluations) were the five studies providing these. We observed sensitivities from 0.682 to 0.964 in these rows. Specificity spread from 0.667 to 0.963. We did not combine these results into a fixed sensitivity/specificity pool (Figure 4). This approach allowed us to track consistency in directionality and examine the results of individual studies more closely.

**Figure 4:**
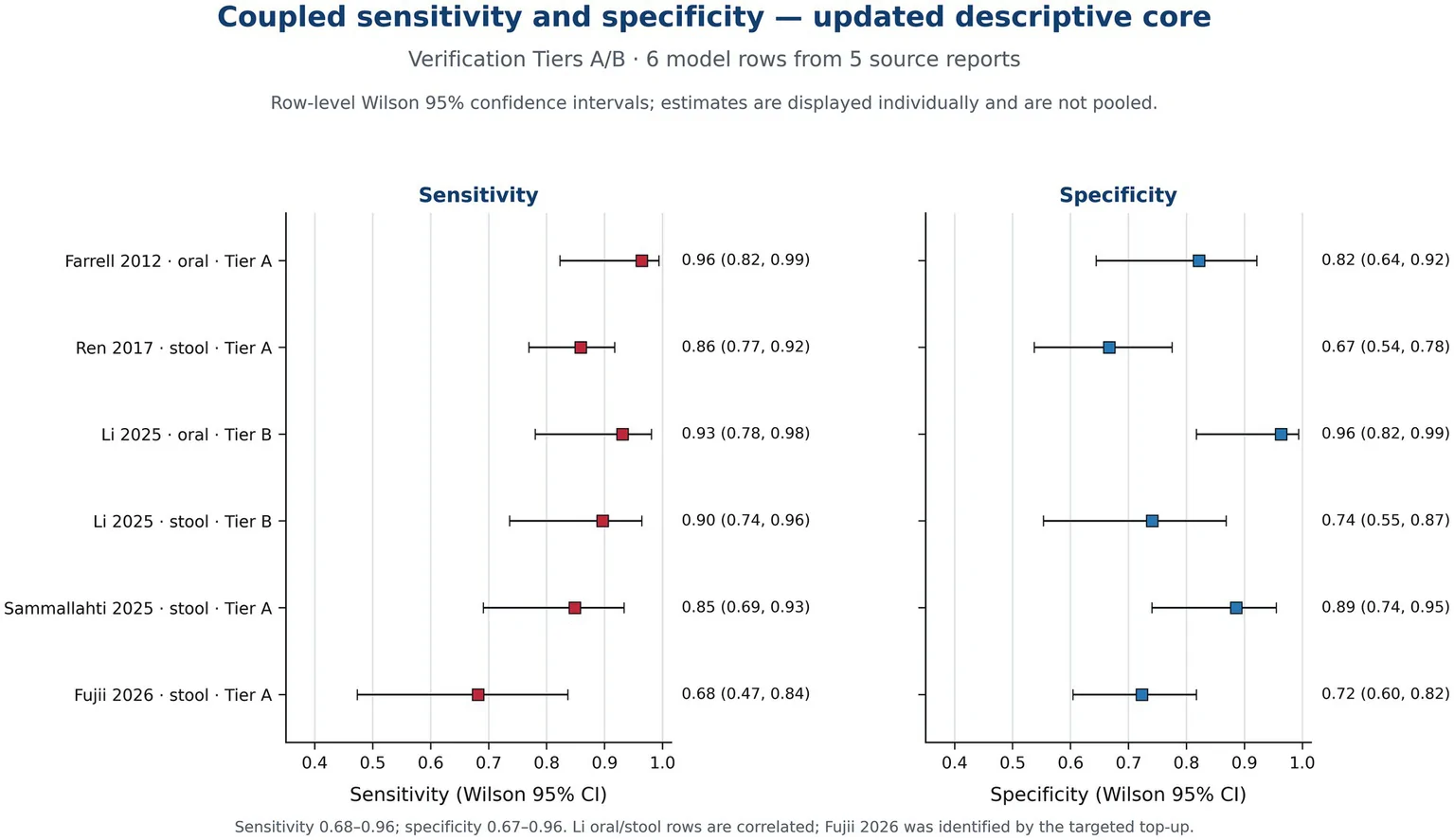
Coupled sensitivity and specificity forest for the updated primary Tier A/B fixed-threshold descriptive core (six model rows from five source reports). Row-level estimates and 95% Wilson confidence intervals are displayed without pooling. Fujii 2026 was identified in the *post-hoc* targeted top-up and is marked accordingly. Excluding it leaves the five-row/four-report original-cutoff core; both layers have very low certainty.

In the analysis of the six-row core dataset, ensuring consistency in the count of study levels is a critical step prior to broader merging. A sequential row-by-row leave-one-out test revealed that no single data row determined the overall descriptive scope; however, this test did not address the within-study dependence arising from paired oral and fecal rows in Li 2025. Based on sample type, we retained the fecal and oral models from Li 2025 independently. Although Fujii 2026 met the Tier A reconstruction criteria, the overall risk of bias remained high because of flaws such as two-gate secondary cohorts, post-selection leave-one-out validation, and threshold optimization using the same data. Given the study-level correlations and risk of bias, this core dataset was only subject to descriptive summarization, and no meta-analysis was conducted.

By constructing a nine-row bivariate model from seven studies, we conducted an analysis from the broader perspective of a fixed-threshold landscape. Here, we further expanded on our core research findings. The two overlapping Chen Y. et al. (2025) rows, the approximate Lu et al. (2019) reconstruction, and the Tier A row from Fujii et al. (2026) were integrated into this approach. Based on the reference exploratory bivariate/HSROC model, sensitivity was 0.86 (95% CI 0.80–0.90) and specificity was 0.80 (95% CI 0.73–0.86; Figure 5). By contrast, after applying separate univariate DerSimonian–Laird models to the same nine rows, the supplementary marginal forests yielded sensitivity 0.83 (95% CI 0.77–0.88) and specificity 0.77 (95% CI 0.71–0.83), respectively (Supplementary Figure S4). Instead of offering a competing reference estimate, the univariate numbers are presented as supporting visualizations to accompany the bivariate/HSROC result, the reference exploratory summary. The complete analytical hierarchy is summarized in Supplementary Table S2. We recognize that the main limitations of this extension are approximate reconstruction, the fact that the nine rows of data come from only seven studies, and the failure to account for within-study correlations. The estimates we derived are intended solely to propose hypotheses, and their value as guidance for clinical practice has not yet been established. Marked row heterogeneity and wide uncertainty intervals support keeping this analysis exploratory; Supplementary Table S5 contains updated summary and sensitivity outputs. In Supplementary Table S5, we outline the negative likelihood ratio, positive likelihood ratio, and diagnostic odds ratio outputs as exploratory ratio measures. This interpretive division was fully intentional. We noted that the broader metrics were directionally consistent; nevertheless, the six-row Tier A/B core remained the primary descriptive subset, was not pooled, and carried a high risk of bias within a very low-certainty evidence base.

**Figure 5:**
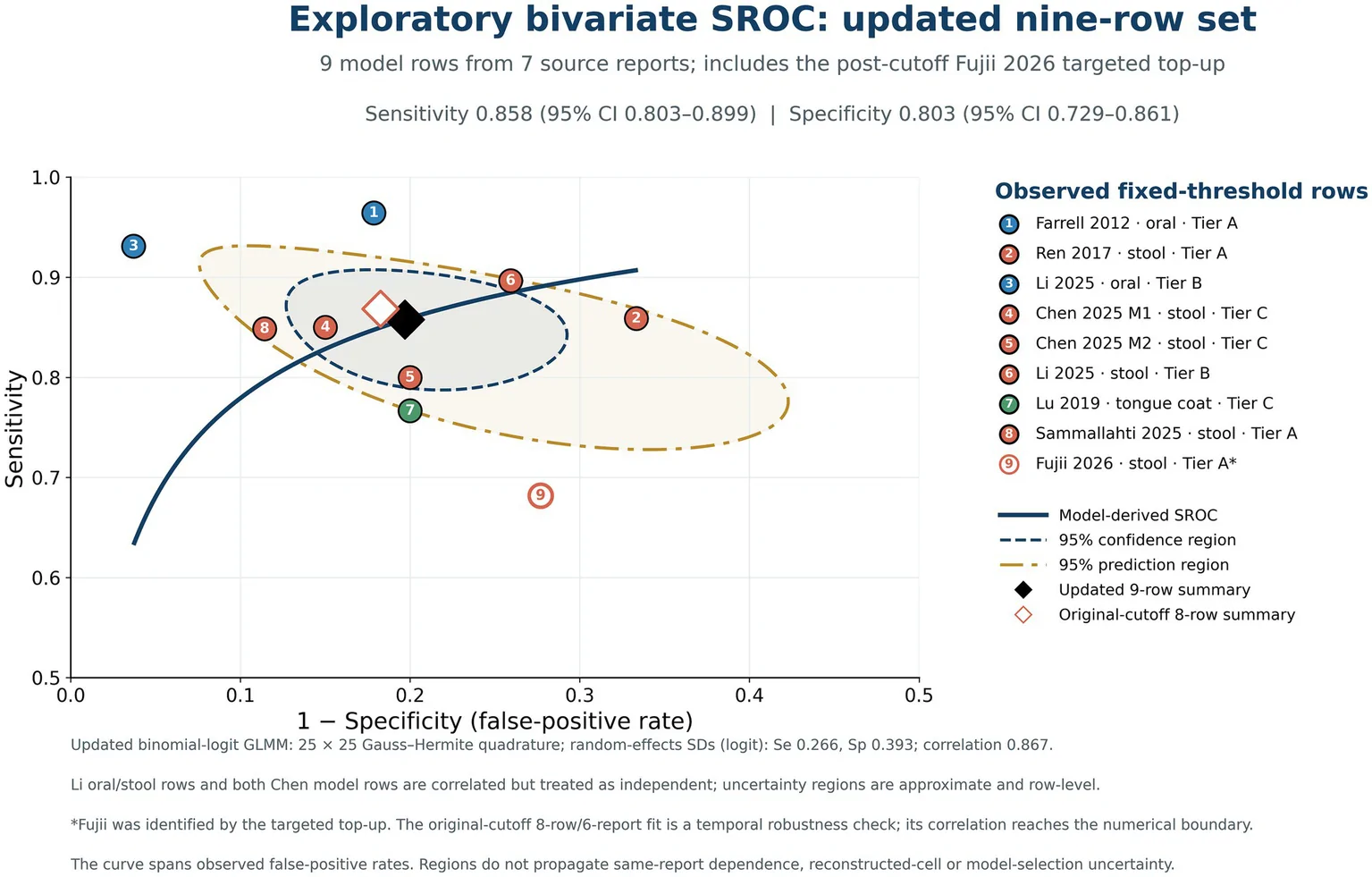
Model-derived SROC for the updated exploratory bivariate synthesis of nine fixed-threshold model rows from seven source reports. The SROC, summary point, 95% confidence region for the fixed effects, and 95% row-level prediction region derive from the executed binomial-logit bivariate random-effects fit. Summary sensitivity is 0.86 (95% CI 0.80–0.90) and specificity 0.80 (0.73–0.86). The fitted correlation is approximately 0.867 and is not at the numerical boundary. Fujii is identified as a post-cutoff targeted-top-up row. Within-report covariance, reconstruction uncertainty, and model-selection uncertainty are not captured by these regions. The original-cutoff eight-row/six-report fit is reported as a temporal sensitivity analysis in Supplementary Tables S2, S5; its correlation reaches the numerical boundary. Separate nine-row univariate DerSimonian–Laird marginal forests are in Supplementary Figure S4. Full statistical definitions and numerical checks are in Supplementary Methods M2.

Consistent with moderate-to-strong discrimination, HSROC points in the expanded model occupied the upper-left region, yet uncertainty remained substantial. For the expanded exploratory model, nine rows represented seven studies, and correlated rows were not modeled directly; confidence and prediction regions therefore do not capture all within-study uncertainty. Reflecting this approach, the six-row Tier A/B core data are displayed solely as study-level estimates accompanied by 95% Wilson CIs; no pooled summary or prediction interval is reported. In our subgroup assessments, oral models stood out for their sensitivity, while the clearest evidence base for external validation was consistently found in stool-based studies. However, the tongue-coat signal is highly provisional, being based on just a single approximate row.

With k remaining below 10 and several data rows derived via approximate reconstruction, a formal interpretation of small-study effects was not warranted, bounding our publication-bias assessment. We provide Deeks’ exploratory output only in Supplementary material.

### AUROC-only discrimination evidence

In constructing the descriptive discrimination inventory for this study, we recorded 51 AUROC estimates categorized into three groups: stool, oral, and tongue-coat domains. Among the 21 sample-domain entries included in our review, 13 lacked stable fixed-threshold 2 × 2 data: 10 were classified as AUROC-only and 3 as exploratory candidates. Supplementary Table S6 and Supplementary Figure S2 catalog the 51 reported AUROC estimates, including multiple overlapping model outputs rather than standalone DTA rows. These AUROC outputs were retained descriptively and excluded from the fixed-threshold DTA synthesis.

This layer also revealed the clearest external-validation evidence for discrimination in stool programs by Kartal et al. (2022), Nagata et al. (2022), and Sammallahti et al. (2025); however, Kartal and Nagata remained in the AUROC-only layer and did not provide stable fixed-threshold 2 × 2 data.

Overall, most models were developed in tertiary settings rather than frontline diagnostic pathways. Since many studies prioritize healthy volunteers over benign confounding lesions like autoimmune pancreatitis, the high AUROC values reported often struggle to reach the status of immediately credible clinical thresholds.

### Stratified evidence across sample domain, comparator structure, and validation maturity

We conducted a systematic stratification of this review by cross-referencing 21 sample-domain entries against three key dimensions: validation maturity, sample domain or technology, and the structure of comparators. See Supplementary Table S1 for the complete evidence map; Figure 6 shows a simplified summary of the subpanels (Panels A–C).

**Figure 6:**
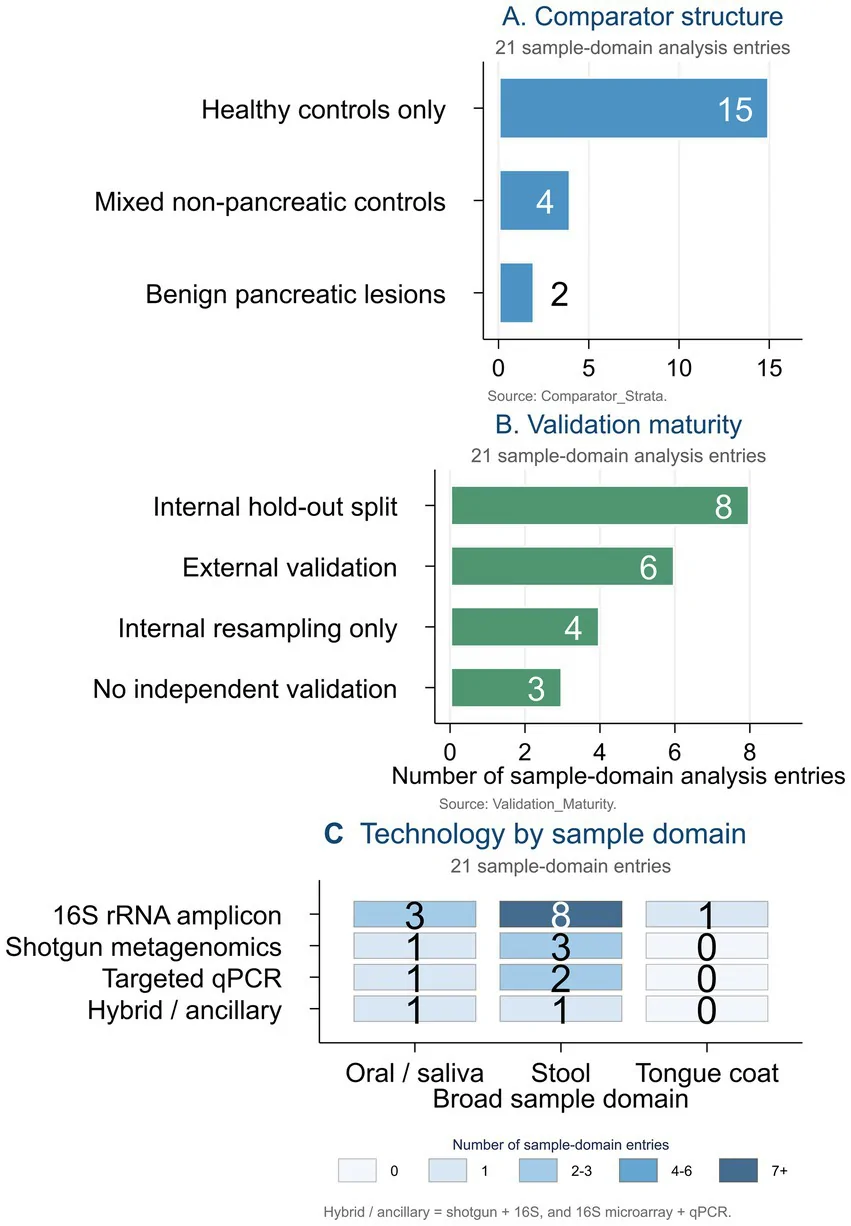
Stratified evidence map for the 21 sample-domain entries. **(A)** Shows comparator structure, **(B)** Validation maturity, and **(C)** The technology-by-sample-domain matrix. Counts refer to sample-domain entries rather than AUROC estimates.

Taken together, these data show that the current comparator design still fails to accurately reflect real-world clinical conditions. Healthy-control contrasts predominated in the sample (15/21 entries), while only 2 entries used benign pancreatic lesions as primary comparators, and another 4 used mixed, non-pancreatic cohorts. Expanding the definition of benign-pancreatic support further revealed that only 2 entries provided a primary contrast, 5 offered secondary support, and 3 included contextual information, with 11 of the 21 entries providing no such support. Note that these statistics pertain to the number of sample-domain entries rather than AUROC estimates at the model level. A major diagnostic challenge currently facing clinicians is how to distinguish PDAC from benign conditions with similar clinical presentations, such as chronic pancreatitis; the practical significance of this differential diagnosis for clinical intervention goes far beyond the simple isolation of healthy controls.

Analysis of the sample sources revealed that they were primarily derived from stool (14/21), followed by saliva/oral cavity (6/21) and tongue coating (1/21). There was also an imbalance in the technologies used: 12 of the 21 entries employed 16S rRNA amplicon sequencing, 4 used shotgun metagenomics, and the remainder used targeted qPCR, hybrid shotgun/16S, or legacy microarray-qPCR; Fujii 2026 employed targeted stool qPCR. Figure 6C shows that shotgun metagenomics was almost exclusively concentrated in stool samples, while non-stool samples primarily relied on 16S-based methods.

After segmenting the validation data, it became clear that there were substantial differences in the maturity levels of the various entries: among the entries reviewed, 6 used external validation, 8 relied on internal hold-out splits, 4 used only internal resampling, and 3 did not perform any independent validation. When reclassified using the evidence-layer framework, six dataset entries formed the primary fixed-threshold core. Tier C evidence within the expanded layer derived from two studies (Lu 2019 and Chen 2025b); since Chen 2025b added two model rows, Tier C consists of three rows in Table 2. Three entries were classified as exploratory candidates, while the remaining 10 were based solely on AUROC. From a clinical perspective, this distinction is crucial, as multiple studies with external validation still omitted a stable 2 × 2 threshold, essentially barring them from any formal pooled DTA synthesis. The audit workbook documents the limited number of studies providing direct early- or resectable-stage support.

**Table 2:** Verification-classified fixed-threshold 2 × 2 analyses used in the primary core and expanded nine-row sensitivity model.

Study/model	Sample	Cases	Controls	2 × 2 (TP/FP/TN/FN)	Sensitivity	Specificity	AUC	Verification / role
Farrell 2012	Oral/saliva	28	28	27/5/23/1	0.964	0.821	0.900	Tier A — primary core
Ren 2017	Stool	85	57	73/19/38/12	0.859	0.667	0.842	Tier A — primary core
Li 2025 [oral]	Oral/saliva	29	27	27/1/26/2	0.931	0.963	0.963	Tier B — primary core
Chen 2025b M1	Stool	20	20	17/3/17/3	0.850	0.850	0.923	Tier C — expanded only
Chen 2025b M2	Stool	20	20	16/4/16/4	0.800	0.800	0.853	Tier C — expanded only
Li 2025 [stool]	Stool	29	27	26/7/20/3	0.897	0.741	0.890	Tier B — primary core
Lu 2019	Tongue coat	30	25	23/5/20/7	0.767	0.800	0.802	Tier C — expanded only
Sammallahti 2025	Stool	33	35	28/4/31/5	0.848	0.886	0.880	Tier A — primary core
Fujii 2026	Stool	22	65	15/18/47/7	0.682	0.723	0.780	Tier A — primary core

Values in this table are reconstructed 2 × 2-derived estimates for DTA analysis; originally reported values, where different because of rounding or validation-split reconstruction, are labelled in Table 1 and the reconstruction log. The two Chen 2025b rows share an overlapping validation cohort and are removed together in the drop-both-Chen sensitivity analysis; the *post-hoc* one-row-per-study analysis also retains only one Li 2025 sample-domain row. Comparator structure for each row (healthy, benign/low-grade, mixed) is given in Supplementary Table S1.

## Discussion

### Principal findings

Our findings indicate that, although 16 studies and 21 sample-domain entries demonstrate the diagnostic potential of noninvasive microbiota-based testing for PDAC, no clinically validated assay has yet been developed in this field, and the current evidence has very low certainty. Importantly, the existing evidence does not yet support a definitive clinical test; rather, a compact core of evidence based on fixed thresholds is currently embedded within a much broader body of literature focused on discrimination. The descriptive literature highlights frequent ecological changes characterized by a slight decrease in diversity, a reduction in commensal bacteria, and pathobiont proliferation in both the oral and stool compartments. From the standpoint of diagnostic test accuracy (DTA), however, making clinically valid inferences depends fundamentally on fixed-threshold data instead of simply pointing to generalized dysbiosis.

Making this specific distinction plays a pivotal role in the underlying methodology. Studies with high AUROC scores serve a distinct objective in model development and signal discovery; however, it is not self-evident that this evidence can be further generalized to clinical diagnoses based on fixed thresholds. To prevent an overestimation of the field’s maturity, we separated our analysis: we distinguished the primary fixed-threshold core rows (6 in total) from the expanded sensitivity rows (9 in total) and the 51 AUROC model-level estimates. This transparent classification strategy prevents us from overstating the clinical maturity of current microbiota testing.

### Clinical interpretation

As shown in Table 2, oral classifiers achieve numerically higher point estimates compared to stool classifiers. However, we assess clinical “maturity” along three specific axes: multi-centre replication, CA19-9 hybrid performance, and external validation within independent cohorts. Currently, stool-based testing frameworks (Kartal et al., 2022; Nagata et al., 2022; Sammallahti et al., 2025) provide the clearest externally validated discrimination evidence; oral evidence remains comparatively thin. In this comparison, “maturity” refers to the external-validation axis rather than stable fixed-threshold classification: Kartal et al. (2022) and Nagata et al. (2022) remain in the AUROC-only layer, whereas Sammallahti et al. (2025) contributes to the A/B core. Stool accounts for the majority of data and utilizes both shotgun and 16S workflows. Heterogeneity across validation approaches and comparator structures remains substantial (Half et al., 2019; Zhou et al., 2021; Kartal et al., 2022; Nagata et al., 2022; Sammallahti et al., 2025; Villani et al., 2025; Fujii et al., 2026). Rather than reflecting random fluctuation, the underlying ecological signal exhibits clear structure. This shift is defined by a selective proliferation of pathobiont taxa—specifically *Fusobacterium*, *Streptococcus*, *Veillonella*, and *Escherichia/Shigella*—coupled with a simultaneous depletion of SCFA-producing commensals. Comparable cross-compartment phenomena have likewise been documented in broader systematic reviews (Wei et al., 2019; Li et al., 2021; Merali et al., 2022).

From a practical standpoint, oral and saliva-based diagnostic methods are highly attractive because they are easy to collect and typically exhibit high sensitivity. However, the strongest estimates currently available are still limited to the framework of case–control studies; furthermore, there is a lack of clear evidence demonstrating their stability when faced with benign pancreatic mimics. Unstimulated saliva, mouthwash, and tongue-coating samples should not be considered equivalent as index tests, as comparative studies based on biological pathways remain scarce. These oral signals remain highly context-dependent (Michaud and Izard, 2014). From a clinical perspective, the directional stability currently observed does not yet demonstrate the consistency required to support definitive clinical applications. Recent studies illustrate both the potential of oral sampling and the ongoing need for pathway-based validation (Farrell et al., 2012; Chen et al., 2023; Li et al., 2024; Li et al., 2025; Tavanaeian et al., 2025; Koopaie et al., 2025). Therefore, oral testing is currently limited to the field of adjunctive triage rather than bedside implementation.

Evidence for tongue-coat microbiota remains exploratory. The Lu et al. (2019) study—currently the only available evidence—suggests that tongue-coating microbiota may offer discriminatory information, yet we cannot establish transportable clinical performance through a single, approximate fixed-threshold reconstruction alone.

AUROC, sensitivity, and specificity merely quantify discriminatory power under specific experimental conditions but do not directly demonstrate clinical utility. Most of the included studies employed a two-gate case–control design and used healthy controls, which introduced spectrum bias and selection bias. This can result in measured performance being better than would be expected against chronic pancreatitis, pancreatic cystic lesions, or autoimmune pancreatitis, or in low-prevalence clinical pathways. It should also be noted that this limitation applies to studies that underwent validation but still used selected controls (Zhou et al., 2021; Li et al., 2024; Li et al., 2025; Sammallahti et al., 2025) (Supplementary Table S1; Figure 6A). Calibration, prevalence-specific predictive values, decision-curve net benefit, and clinical-impact outcomes were generally unavailable. Under-reporting of contamination control and batch-effect handling further limits transportability. These classifiers should therefore be regarded as investigational adjuncts for risk stratification or diagnostic triage rather than validated population-screening tests.

Clearly distinguishing between different levels of validation can reduce the risk of overestimating diagnostic maturity. For this reason, our manuscript explicitly separates the six-row core, the three additional approximate reconstructions, and the AUROC-only layer.

### Methodologic limitations of current evidence

In terms of actual clinical translation, one should not expect microbiota assays to operate as independent mechanisms for population-wide screening anytime in the near term. Instead, they will likely first emerge as minimally invasive, supplementary triage instruments. Such use is most appropriate within symptomatic diagnostic pathways or, investigationally, alongside CA19-9 and EUS/MRI surveillance of high-risk individuals, rather than as population screening. Ultimately, rather than debating universal screening, clinicians must determine the degree to which an abnormal oral or stool finding increases diagnostic probability prior to ordering biopsies or scans. Here, serial sampling of saliva or feces may prove particularly useful—assuming upcoming longitudinal studies confirm that microbial signatures change prior to formal disease detection. Furthermore, achieving clinically actionable triage will almost certainly require broad multifeature panels rather than relying on individual microbial strains (Tijeras-Raballand et al., 2021; Miyabayashi et al., 2022).

The inferential weight of this review is subject to specific constraints, detailed here in decreasing order of importance. The primary issue is that standard case–control cohorts, rather than diagnostic pathways, supply most of the fixed-threshold evidence. Next, the expanded synthesis included three approximate Tier C rows and comprised nine rows from only seven studies. Participant-level covariance was unavailable, so within-study dependence could not be modeled and the nine-row confidence intervals may be too narrow; the one-row-per-study analysis reduced, but did not eliminate, uncertainty arising from small k and deterministic row selection. A third challenge pertains to the choice of comparators; the current literature is heavily skewed toward healthy-control contrasts, which means that benign pancreatic conditions remain notably underrepresented in most studies. Methodological disparities present a fourth challenge. Pre-analytic factors (sampling protocol, storage, and recent antibiotic, PPI, or probiotic exposure), analytic factors (DNA extraction, sequencing platform or target, bioinformatic filtering, contamination controls, and batch correction), and population factors (geography, smoking, and metabolic profile) can each shift microbial abundance and model thresholds (Ammer-Herrmenau et al., 2020; Panebianco et al., 2022; Sexton et al., 2022). Reporting was too sparse and heterogeneous for reliable meta-regression, so these effects could not be quantitatively separated and the exploratory pooled estimates should not be assumed transportable across laboratories or populations. Lastly, researchers predominantly employ single-time-point sampling near diagnosis and rely heavily on internal or semi-external validation. With the prediagnostic window and cross-setting transportability still unresolved, the main conclusion should be interpreted conservatively.

Moving past the simple cataloging of dysbiosis, the discipline now prioritizes noninvasive classifiers with tangible potential for clinical diagnosis. However, the collective evidence currently represents a developing DTA signal, not a fully validated clinical test (Santos Cruz et al., 2024; Chen X. et al., 2025; Daniel et al., 2025; Suresh et al., 2025).

### Implications for future validation

Based on the conclusions of this review, to truly bring microbiota diagnostics into clinical practice in the future, the following key design considerations must be addressed. First, patient recruitment can no longer rely on “manually selected cases and healthy controls”; instead, patients must be identified directly within real-world hospital care pathways. Second, data aggregation and analysis must follow established protocols; it is not sufficient to provide only a bare AUROC value—sensitivity, specificity, and a specific 2 × 2 table at a fixed threshold must be clearly presented. Third, comparators must include patients with benign pancreatic mimics to determine whether the method can accurately distinguish them. Fourth, the entire process demands standardization, aligning sequencing techniques and bioinformatic analysis directly with patient staging systems and clinical outcome milestones. Fifth, patients should undergo serial sampling whenever possible to quantify the prediagnostic window. Finally, when developing the model, use multi-taxon panels rather than relying on a single taxon; this may help the technique be replicated in other laboratories or regions.

Our current findings suggest that integrated analytical approaches are likely to outperform analytical platforms that rely solely on microbial data (Huang et al., 2021; Thomas, 2025). In line with overarching trends in biomarker discovery, panel-based approaches deliver peak clinical performance when they integrate nascent biological signals with CA19-9, particularly when evaluated against precursor states or chronic pancreatitis rather than strictly healthy baselines. A small number of previous studies have tested these microbial profiles in combination with standard clinical markers or CA19-9. Whenever implemented, such multimodal frameworks generally exceeded the performance of standalone microbiota assessments, although limitations related to semi-external or internal validation remain (Kartal et al., 2022; Nagata et al., 2022; Li et al., 2025). It will be important for upcoming literature to clearly separate basic joint-negativity or either-positive algorithms from comprehensive multivariable architectures, as each strategy balances sensitivity and specificity differently.

The structured DTA synthesis clarified several requirements for future clinical translation. However, its clinical application depends on studies that are externally validated, methodologically consistent, fixed-threshold, comparator-realistic, and stage-aware. The very low certainty of the current evidence defines the need for further research. Since this signal is biologically plausible but methodologically fragile, the field requires the rigorous, externally validated, and fixed-threshold studies that we have outlined above. Mapping these gaps is one of the main contributions of this report.

Reviews exploring the mechanistic and clinical dimensions of the PDAC microbiome regularly highlight an ecological disruption marked by clear compartment-specific profiles across biliary, intratumoral, oral, and intestinal niches. Although these papers highlight potential translational implications, they also frequently point out stubborn methodological obstacles (Panebianco and Pazienza, 2019; Herremans et al., 2022). By providing a useful ecological perspective, the body of existing work helps frame both our AUROC-only evidence layer and the k = 9 exploratory model—adding context to this DTA synthesis. However, general contextual reviews cannot replace the need for externally validated, threshold-defined DTA data, for which current evidence remains limited.

A growing body of mechanistic and translational research indicates that microbial migration along the oral-gut-pancreatic axis, chronic inflammation, barrier dysfunction, immune remodeling, metabolite signaling, and their interactions with chemotherapy and immunotherapy may be central to this process. However, as repeatedly emphasized in the relevant literature, methodological heterogeneity and case–control enrichment remain the primary obstacles hindering immediate clinical translation (Zambirinis et al., 2014; de Castilhos et al., 2024).

Recent reviews of PDAC biomarkers and liquid biopsy place emerging noninvasive signals within multimarker and multi-omics frameworks, while emphasizing the need for standardized methods and prospective clinical validation before routine implementation (Yu et al., 2025; Bao et al., 2024). In this context, microbiota classifiers may be better investigated as adjuncts to CA19-9, liquid-biopsy, metabolomic, imaging, and clinical variables than as stand-alone population-screening tests. Clinical implementation still requires validation that is externally transportable, comparator-realistic, and stage-aware (Ahmed et al., 2024; Bengtsson et al., 2025; Murray et al., 2025; Bogdanski et al., 2025).

When interpreted within an adjunctive, pathway-oriented framework, these findings align closely with the current consensus guidelines governing pancreatic research. Documents from the AGA, ASGE, and CAPS support EUS/MRI-based surveillance only for carefully selected high-risk individuals while explicitly opposing the use of biomarkers alone. The current guidelines from the Japan Pancreas Society and the CSCO follow a similar approach, deeply integrating diagnostic procedures into a pathology-centered, multidisciplinary imaging workflow (Goggins et al., 2020; Aslanian et al., 2020; Sawhney et al., 2022; Cui et al., 2022; Abe et al., 2026). Meanwhile, several recent statements on microbiome contamination control and best practices have repeatedly emphasized crucial logistical standards, including transparency in reporting, complete documentation of metadata, the establishment of negative controls, disciplined storage, contamination-aware sampling, and standardization of terminology. Whether PDAC microbial classifiers can achieve clinical transportability will require harmonization across these domains (Isali et al., 2024; Fierer et al., 2025).

## Conclusion

Current evidence provides a biologically plausible but very low-certainty diagnostic signal. Noninvasive microbiota-based PDAC classifiers should not be read as supporting stand-alone clinical use. The most informative subset comprised six fixed-threshold rows from five studies; all evaluated models carried high overall QUADAS-2 risk of bias, and expanded estimates remained exploratory sensitivity analyses rather than primary clinical benchmarks. Stool-based programs provide the clearest external-validation evidence for discrimination, although Kartal et al. (2022) and Nagata et al. (2022) remain AUROC-only and fixed-threshold evidence is sparse; oral sampling remains attractive on operational grounds: it is well tolerated, easily repeated, and compatible with precision-medicine triage pathways. Future validation should therefore prespecify the microbial panel, define explicit fusion rules with CA19-9, and demonstrate discrimination from chronic pancreatitis and other benign pancreatic mimics. Standardization of oral sampling, stage-aware multicenter testing, and prospective serial sampling are required to determine whether such microbial signals add clinically meaningful lead time over standard diagnostic pathways.

## Data Availability

Study-level tables, reconstructed 2×2 cells, and analytical summaries are provided in the Supplementary material. The accompanying author package contains the extraction workbook, source and decision logs available to the authors, executed Python analysis code, input snapshots, numerical outputs, and figure-generation files. Files from the submitted version are publicly available at https://osf.io/h358f/ (DOI: https://doi.org/10.17605/OSF.IO/H358F). The supplied materials do not establish a complete line-byline log of all 19 targeted top-up screening decisions.
