# Supplementary material for: Noninvasive microbiota-based diagnostic models for pancreatic ductal adenocarcinoma: a systematic review and diagnostic test accuracy meta-analysis
Source: Front Microbiol. 2026 Sep 23;17:1866794. doi: 10.3389/fmicb.2026.1866794 (PMC13644726; doi:10.3389/fmicb.2026.1866794)
Supplement: Supplementary file 1 [file Supplementary_file_1.docx]

**PRISMA 2020 Checklist - Completed Standard Version**

**Noninvasive microbiota-based diagnostic models for pancreatic ductal adenocarcinoma: a systematic review and diagnostic test accuracy meta-analysis**

Authors: Liang Zhang, Jian Liu, Mengyao Pang, Chenfei Liang, Da Li, Yali Wang | Target journal: Frontiers in Microbiology | Prepared: September 6, 2026

Note. Locations refer to the current Reviewer 4 revision of the main manuscript and the accompanying Supplementary Material and reproducibility files. This standard PRISMA 2020 checklist complements, but does not replace, the PRISMA-DTA checklist for diagnostic test accuracy reviews.

| **Section and Topic** | **Item #** | **Checklist item** | **Location where item is reported** |
| --- | --- | --- | --- |
| **TITLE** | **TITLE** | **TITLE** |  |
| Title | **1** | Identify the report as a systematic review. | Title page (manuscript title). |
| **ABSTRACT** | **ABSTRACT** | **ABSTRACT** |  |
| Abstract | **2** | See the PRISMA 2020 for Abstracts checklist. | Single-paragraph Abstract with structured labels; 300 words by whitespace count. |
| **INTRODUCTION** | **INTRODUCTION** | **INTRODUCTION** |  |
| Rationale | **3** | Describe the rationale for the review in the context of existing knowledge. | Introduction. |
| Objectives | **4** | Provide an explicit statement of the objective(s) or question(s) the review addresses. | Introduction (review objective and evidence-layer framework). |
| **METHODS** | **METHODS** | **METHODS** |  |
| Eligibility criteria | **5** | Specify the inclusion and exclusion criteria for the review and how studies were grouped for the syntheses. | Methods—Eligibility criteria. |
| Information sources | **6** | Specify all databases, registers, websites, organisations, reference lists and other sources searched or consulted to identify studies. Specify the date when each source was last searched or consulted. | Methods—Search strategy and review layers; Supplementary Table S3. The original eight-database search and targeted PubMed/Europe PMC top-up are reported separately. |
| Search strategy | **7** | Present the full search strategies for all databases, registers and websites, including any filters and limits used. | Methods—Search strategy and review layers; Supplementary Table S3; audit workbook. The top-up sheet gives reproducible concepts and aggregate yields but does not enumerate all 19 screened reports line by line. |
| Selection process | **8** | Specify the methods used to decide whether a study met the inclusion criteria of the review, including how many reviewers screened each record and each report retrieved, whether they worked independently, and if applicable, details of automation tools used in the process. | Methods—Data extraction and evidence structuring; Generative AI statement; Supplementary Methods M7; Figure 1; Supplementary Table S3. Final audit checked source reports, but reviewer-level records were unavailable; independent duplicate screening/adjudication is not claimed. |
| Data collection process | **9** | Specify the methods used to collect data from reports, including how many reviewers collected data from each report, whether they worked independently, any processes for obtaining or confirming data from study investigators, and if applicable, details of automation tools used in the process. | Methods—Data extraction and evidence structuring; Generative AI statement; Supplementary Methods M7; Supplementary Tables S1A–S6 and audit workbook. Reviewer-level records were unavailable; independent duplicate extraction is not claimed. |
| Data items | 10a | List and define all outcomes for which data were sought. Specify whether all results that were compatible with each outcome domain in each study were sought (e.g. for all measures, time points, analyses), and if not, the methods used to decide which results to collect. | Methods—Data extraction and evidence structuring and 2×2 reconstruction rules; Tables 1–2; Supplementary Tables S2 and S4–S6. |
| Data items | 10b | List and define all other variables for which data were sought (e.g. participant and intervention characteristics, funding sources). Describe any assumptions made about any missing or unclear information. | Methods—Eligibility criteria and Data extraction and evidence structuring; Supplementary Tables S1A–S1B. NR is distinguished from absence. |
| Study risk of bias assessment | **11** | Specify the methods used to assess risk of bias in the included studies, including details of the tool(s) used, how many reviewers assessed each study and whether they worked independently, and if applicable, details of automation tools used in the process. | Methods—risk-of-bias description; Generative AI statement; Supplementary Methods M7; Figure 3. Judgments are shown for 20 model-based records; independent duplicate assessment is not claimed. |
| Effect measures | **12** | Specify for each outcome the effect measure(s) (e.g. risk ratio, mean difference) used in the synthesis or presentation of results. | Methods—2×2 reconstruction rules and Statistical analysis; sensitivity, specificity, AUROC, LR+, LR−, and DOR definitions. |
| Synthesis methods | 13a | Describe the processes used to decide which studies were eligible for each synthesis (e.g. tabulating the study intervention characteristics and comparing against the planned groups for each synthesis (item #5)). | Methods—Data extraction and evidence structuring and Statistical analysis; Tables 1–2; Supplementary Table S2 (row/report membership). |
| Synthesis methods | 13b | Describe any methods required to prepare the data for presentation or synthesis, such as handling of missing summary statistics, or data conversions. | Methods—2×2 reconstruction rules and Statistical analysis; Supplementary Table S4 (sources, tiers, and conditional-interval limitations). |
| Synthesis methods | 13c | Describe any methods used to tabulate or visually display results of individual studies and syntheses. | Methods—Statistical analysis; Figures 4–5 and Supplementary Figures S2–S5. |
| Synthesis methods | 13d | Describe any methods used to synthesize results and provide a rationale for the choice(s). If meta-analysis was performed, describe the model(s), method(s) to identify the presence and extent of statistical heterogeneity, and software package(s) used. | Methods—Statistical analysis; updated nine-row/seven-report bivariate model and original-cutoff eight-row/six-report temporal sensitivity model, and separately labelled marginal models. Supplementary Methods M2 gives actual Python versions, likelihood/optimization, SROC and uncertainty construction, the univariate origin of marginal τ²/I², and an independent R package check with model-specific qualifications; Stata companion code was not executed. |
| Synthesis methods | 13e | Describe any methods used to explore possible causes of heterogeneity among study results (e.g. subgroup analysis, meta-regression). | Methods—structured narrative transportability assessment; Supplementary Tables S1A–S1B. No meta-regression was performed because reporting and independent-report counts were insufficient. |
| Synthesis methods | 13f | Describe any sensitivity analyses conducted to assess robustness of the synthesized results. | Methods—Statistical analysis; one-row-per-source-report and drop-both-Chen analyses; Supplementary Tables S2 and S5. |
| Reporting bias assessment | **14** | Describe any methods used to assess risk of bias due to missing results in a synthesis (arising from reporting biases). | Methods—Statistical analysis; Supplementary Figure S5. Deeks analysis is explicitly exploratory and non-interpretable with eight pre-cutoff rows from six source reports. |
| Certainty assessment | **15** | Describe any methods used to assess certainty (or confidence) in the body of evidence for an outcome. | Methods—adapted, GRADE-informed certainty appraisal; Figure 2. The appraisal is not presented as a formally validated GRADE-DTA tool. |
| **RESULTS** | **RESULTS** | **RESULTS** |  |
| Study selection | 16a | Describe the results of the search and selection process, from the number of records identified in the search to the number of studies included in the review, ideally using a flow diagram. | Results—Study selection and evidence layers; Figure 1; Supplementary Table S3. PRISMA count: 16 included reports/publications, not 16 independent cohorts. |
| Study selection | 16b | Cite studies that might appear to meet the inclusion criteria, but which were excluded, and explain why they were excluded. | Figure 1 (aggregate reasons); Supplementary Table S3 (two detailed top-up exclusions); original full-text exclusion log in the audit workbook. |
| Study characteristics | **17** | Cite each included study and present its characteristics. | Results—Study characteristics; Table 1; Supplementary Tables S1A–S1B (16 reports; 21 sample-domain entries). |
| Risk of bias in studies | **18** | Present assessments of risk of bias for each included study. | Results—Risk of bias and applicability; Figure 3. The plotted unit is 20 model-based records; Table S1A identifies the Tavanaeian stool entry omitted from that set. |
| Results of individual studies | **19** | For all outcomes, present, for each study: (a) summary statistics for each group (where appropriate) and (b) an effect estimate and its precision (e.g. confidence/credible interval), ideally using structured tables or plots. | Results—individual fixed-threshold estimates; Table 2; Figure 4; Supplementary Figures S3–S4 and Table S4 (nine rows reported individually). |
| Results of syntheses | 20a | For each synthesis, briefly summarise the characteristics and risk of bias among contributing studies. | Results—updated six-row/five-report descriptive core and nine-row/seven-report exploratory set; original-cutoff 5/4 and 8/6 temporal subsets in Supplementary Methods M1 and Tables S2/S5; Figures 4–6; Supplementary Tables S1A–S2. |
| Results of syntheses | 20b | Present results of all statistical syntheses conducted. If meta-analysis was done, present for each the summary estimate and its precision (e.g. confidence/credible interval) and measures of statistical heterogeneity. If comparing groups, describe the direction of the effect. | Results—exploratory bivariate and dependency-sensitivity estimates with 95% CIs; Supplementary Tables S2 and S5. Figure 5 reports model-derived confidence and row-level prediction regions conditional on row independence; no within-report covariance correction is claimed. |
| Results of syntheses | 20c | Present results of all investigations of possible causes of heterogeneity among study results. | Results—comparator, validation, technology, and transportability assessments; Figure 6; Supplementary Tables S1A–S1B. |
| Results of syntheses | 20d | Present results of all sensitivity analyses conducted to assess the robustness of the synthesized results. | Results—one-row-per-source-report (7/7) and drop-both-Chen (7/6) analyses; Supplementary Tables S2 and S5. |
| Reporting biases | **21** | Present assessments of risk of bias due to missing results (arising from reporting biases) for each synthesis assessed. | Supplementary Figure S5; explicitly non-interpretable for publication-bias inference (eight pre-cutoff rows from six source reports). |
| Certainty of evidence | **22** | Present assessments of certainty (or confidence) in the body of evidence for each outcome assessed. | Results—adapted, GRADE-informed certainty appraisal; Figure 2. |
| **DISCUSSION** | **DISCUSSION** | **DISCUSSION** |  |
| Discussion | 23a | Provide a general interpretation of the results in the context of other evidence. | Discussion—Principal findings and Clinical interpretation; discrimination is distinguished from clinical utility and placed in the multimodal-biomarker context. |
| Discussion | 23b | Discuss any limitations of the evidence included in the review. | Discussion—Clinical interpretation and Methodologic limitations; dependence, comparator bias, reconstruction uncertainty, and microbiome-specific transportability. |
| Discussion | 23c | Discuss any limitations of the review processes used. | Discussion—Methodologic limitations; Supplementary Methods M1–M3 and M7 (small effective report count, post hoc analyses and incomplete workflow records). |
| Discussion | 23d | Discuss implications of the results for practice, policy, and future research. | Discussion—Implications for future validation; adjunctive triage/high-risk surveillance and multimodal validation. |
| **OTHER INFORMATION** | **OTHER INFORMATION** | **OTHER INFORMATION** |  |
| Registration and protocol | 24a | Provide registration information for the review, including register name and registration number, or state that the review was not registered. | Methods—Study design and registration; PROSPERO CRD420261370926. |
| Registration and protocol | 24b | Indicate where the review protocol can be accessed, or state that a protocol was not prepared. | Methods—Study design and registration; PROSPERO/OSF locations in Supplementary Material. |
| Registration and protocol | 24c | Describe and explain any amendments to information provided at registration or in the protocol. | Methods—Study design and registration; Supplementary Methods M1 (post hoc updated membership and reconstruction tiers). Formal protocol-amendment records require author verification in PROSPERO/OSF. |
| Support | **25** | Describe sources of financial or non-financial support for the review, and the role of the funders or sponsors in the review. | Funding statement. |
| Competing interests | **26** | Declare any competing interests of review authors. | Conflict of interest statement. |
| Availability of data, code and other materials | **27** | Report which of the following are publicly available and where they can be found: template data collection forms; data extracted from included studies; data used for all analyses; analytic code; any other materials used in the review. | Data availability statement; Supplementary Material; analysis kit/audit files. The submitted-version files are publicly accessible; proof-stage corrections are supplied separately. |
