# Supplementary material for: Noninvasive microbiota-based diagnostic models for pancreatic ductal adenocarcinoma: a systematic review and diagnostic test accuracy meta-analysis
Source: Front Microbiol. 2026 Sep 23;17:1866794. doi: 10.3389/fmicb.2026.1866794 (PMC13644726; doi:10.3389/fmicb.2026.1866794)
Supplement: Supplementary file 2 [file Supplementary_file_2.docx]

| **Section/topic** | **#** | **PRISMA-DTA Checklist Item** | **Reported on page #** |
| --- | --- | --- | --- |
| **TITLE / ABSTRACT** | | |  |
| Title | 1 | Identify the report as a systematic review (+/- meta-analysis) of diagnostic test accuracy (DTA) studies. | Title page (manuscript title). |
| Abstract | 2 | Abstract: See PRISMA-DTA for abstracts. | Single-paragraph Abstract with structured labels; 300 words by whitespace count. |
| **INTRODUCTION** | | |  |
| Rationale | 3 | Describe the rationale for the review in the context of what is already known. | Introduction. |
| Clinical role of index test | D1 | State the scientific and clinical background, including the intended use and clinical role of the index test, and if applicable, the rationale for minimally acceptable test accuracy (or minimum difference in accuracy for comparative design). | Introduction; Methods—Eligibility criteria; Discussion—Clinical interpretation and future validation (adjunctive triage/high-risk surveillance, not population screening). |
| Objectives | 4 | Provide an explicit statement of question(s) being addressed in terms of participants, index test(s), and target condition(s). | Introduction (objective and evidence-layer framework). |
| **METHODS** | | |  |
| Protocol and registration | 5 | Indicate if a review protocol exists, if and where it can be accessed (e.g., Web address), and, if available, provide registration information including registration number. | Methods—Study design and registration; PROSPERO CRD420261370926; repository locations in Supplementary Material. |
| Eligibility criteria | 6 | Specify study characteristics (participants, setting, index test(s), reference standard(s), target condition(s), and study design) and report characteristics (e.g., years considered, language, publication status) used as criteria for eligibility, giving rationale. | Methods—Eligibility criteria. |
| Information sources | 7 | Describe all information sources (e.g., databases with dates of coverage, contact with study authors to identify additional studies) in the search and date last searched. | Methods—Search strategy and review layers; Supplementary Table S3 (original search through 19 Apr 2026; targeted top-up through 3 Sep 2026). |
| Search | 8 | Present full search strategies for all electronic databases and other sources searched, including any limits used, such that they could be repeated. | Methods—Search strategy and review layers; Supplementary Table S3; audit workbook. The supplied top-up sheet reports concepts/aggregate yields but not a line-by-line list of all 19 screened reports. |
| Study selection | 9 | State the process for selecting studies (i.e., screening, eligibility, included in systematic review, and, if applicable, included in the meta-analysis). | Methods—Data extraction and evidence structuring; Supplementary Methods M7; Figure 1; Supplementary Table S3. Reviewer-level workflow records were unavailable; independent duplicate screening/adjudication is not claimed. |
| Data collection process | 10 | Describe method of data extraction from reports (e.g., piloted forms, independently, in duplicate) and any processes for obtaining and confirming data from investigators. | Methods—Data extraction and evidence structuring; Supplementary Methods M7; Supplementary Tables S1A–S6/audit workbook. Independent duplicate extraction is not claimed because reviewer-level records were unavailable. |
| Definitions for data extraction | 11 | Provide definitions used in data extraction and classifications of target condition(s), index test(s), reference standard(s) and other characteristics (e.g. study design, clinical setting). | Methods—Eligibility criteria, Data extraction and evidence structuring, and 2×2 reconstruction rules; Tables 1–2; Supplementary Tables S1A–S1B and S4. |
| Risk of bias and applicability | 12 | Describe methods used for assessing risk of bias in individual studies and concerns regarding the applicability to the review question. | Methods—risk-of-bias description; Supplementary Methods M7; Figure 3. The unit is 20 model-based records; independent duplicate assessment is not claimed. |
| Diagnostic accuracy measures | 13 | State the principal diagnostic accuracy measure(s) reported (e.g. sensitivity, specificity) and state the unit of assessment (e.g. per-patient, per-lesion). | Methods—2×2 reconstruction rules and Statistical analysis; patient-level case/control counts where reconstructable; conditional-interval limitations in Supplementary Table S4. |
| Synthesis of results | 14 | Describe methods of handling data, combining results of studies and describing variability between studies. This could include, but is not limited to: a) handling of multiple definitions of target condition. b) handling of multiple thresholds of test positivity, c) handling multiple index test readers, d) handling of indeterminate test results, e) grouping and comparing tests, f) handling of different reference standards | Methods—Statistical analysis; handling of correlated rows, reconstruction tiers, thresholds, comparators, validation, and one-row-per-source-report sensitivity analysis. |

Checklist section 1 of 2

| **Section/topic** | **#** | **PRISMA-DTA Checklist Item** | **Reported on page #** |
| --- | --- | --- | --- |
| Meta-analysis | D2 | Report the statistical methods used for meta-analyses, if performed. | Methods—Statistical analysis; nine-row/seven-source-report exploratory bivariate model, dependency analyses, and separately labelled univariate marginal models. Supplementary Methods M2 gives actual Python versions, likelihood/optimization, SROC and uncertainty construction, the univariate origin of marginal τ²/I², and an independent R package check with model-specific qualifications; Stata companion code was not executed. |
| Additional analyses | 16 | Describe methods of additional analyses (e.g., sensitivity or subgroup analyses, meta-regression), if done, indicating which were pre-specified. | Methods—Statistical analysis; post hoc one-row-per-source-report and drop-both-Chen analyses; structured narrative transportability assessment. |
| **RESULTS** | | |  |
| Study selection | 17 | Provide numbers of studies screened, assessed for eligibility, included in the review (and included in meta-analysis, if applicable) with reasons for exclusions at each stage, ideally with a flow diagram. | Results—Study selection and evidence layers; Figure 1; Supplementary Table S3 (16 included reports/publications, not 16 independent cohorts). |
| Study characteristics | 18 | For each included study provide citations and present key characteristics including: a) participant characteristics (presentation, prior testing), b) clinical setting, c) study design, d) target condition definition, e) index test, f) reference standard, g) sample size, h) funding sources | Results—Study characteristics; Table 1; Supplementary Tables S1A–S1B (16 reports; 21 sample-domain entries). |
| Risk of bias and applicability | 19 | Present evaluation of risk of bias and concerns regarding applicability for each study. | Results—Risk of bias and applicability; Figure 3 (20 model-based records). Table S1A identifies the Tavanaeian stool entry omitted from that plotted set. |
| Results of individual studies | 20 | For each analysis in each study (e.g. unique combination of index test, reference standard, and positivity threshold) report 2x2 data (TP, FP, FN, TN) with estimates of diagnostic accuracy and confidence intervals, ideally with a forest or receiver operator characteristic (ROC) plot. | Results—primary fixed-threshold core and expanded analyses; Table 2; Figure 4; Supplementary Figures S3–S4 and Table S4 (nine rows). |
| Synthesis of results | 21 | Describe test accuracy, including variability; if meta-analysis was done, include results and confidence intervals. | Results—updated nine-row/seven-report synthesis and variability; original-cutoff sensitivity in Supplementary Methods M1–M3 and Table S5; Figures 4–5; Supplementary Tables S2 and S5. Figure 5 reports model-derived confidence and row-level prediction regions conditional on row independence; no within-report covariance correction is claimed. |
| Additional analysis | 23 | Give results of additional analyses, if done (e.g., sensitivity or subgroup analyses, meta-regression; analysis of index test: failure rates, proportion of inconclusive results, adverse events). | Results—one-row-per-source-report, drop-both-Chen, AUROC, comparator, validation, and transportability analyses; Figure 6; Supplementary Tables S1A–S6. |
| **DISCUSSION** | | |  |
| Summary of evidence | 24 | Summarize the main findings including the strength of evidence. | Results—adapted, GRADE-informed certainty appraisal; Figure 2; Discussion. PRISMA-DTA item 24 is a reporting item, not the appraisal method. |
| Limitations | 25 | Discuss limitations from included studies (e.g. risk of bias and concerns regarding applicability) and from the review process (e.g. incomplete retrieval of identified research). | Discussion—Clinical interpretation and Methodologic limitations; dependence, reconstruction uncertainty, spectrum bias, and microbiome-specific transportability. |
| Conclusions | 26 | Provide a general interpretation of the results in the context of other evidence. Discuss implications for future research and clinical practice (e.g. the intended use and clinical role of the index test). | Conclusions; intended role restricted to investigational adjunctive triage/high-risk surveillance rather than population screening. |
| **FUNDING** | | |  |
| Funding | 27 | For the systematic review, describe the sources of funding and other support and the role of the funders. | Funding statement. |

*Adapted From:* McInnes MDF, Moher D, Thombs BD, McGrath TA, Bossuyt PM, The PRISMA-DTA Group (2018). Preferred Reporting Items for a Systematic Review and Meta-analysis of Diagnostic Test Accuracy Studies: The PRISMA-DTA Statement. JAMA. 2018 Jan 23;319(4):388-396. doi: 10.1001/jama.2017.19163.

Checklist section 2 of 2
