# Supplementary material for: Noninvasive microbiota-based diagnostic models for pancreatic ductal adenocarcinoma: a systematic review and diagnostic test accuracy meta-analysis
Source: Front Microbiol. 2026 Sep 23;17:1866794. doi: 10.3389/fmicb.2026.1866794 (PMC13644726; doi:10.3389/fmicb.2026.1866794)
Supplement: Supplementary file 3 [file Supplementary_file_3.docx]

**Supplementary Material**

# **Contents and denominator hierarchy**

The denominator and analysis-unit hierarchy below is used throughout this Supplementary Material, the main manuscript, figures, tables, and audit files.

**• 16 included reports/publications — the PRISMA report count after the targeted top-up. This is not an independent-cohort count. Fujii 2026 uses previously characterised linked cohorts and contains overlapping subgroup outputs; it is counted as one report and one sample-domain entry, not as multiple independent cohorts.**

**• 20 model-based records — the plotted QUADAS-2/PROBAST unit, not an independent-study count. The Tavanaeian 2025 stool entry remains in the 21-entry descriptive map but is omitted from the plotted risk-of-bias set because its published sensitivity, specificity, and accuracy fields were internally inconsistent and did not support a stable model-based row. Five overall PROBAST judgments coded as unclear were corrected to high because their analysis-domain judgments were high; overall risk is therefore high in all 20 plotted records. Domain-level judgments and fixed-threshold 2×2 inputs are unchanged.**

**• 21 sample-domain entries — the descriptive stratification unit (oral/saliva, stool, and tongue coat); multiple entries may arise from the same source report.**

**• 9 fixed-threshold model rows from 7 source reports — the updated exploratory synthesis. Eight rows from six source reports were available at the original cutoff; Fujii 2026 adds one post-cutoff row through the targeted top-up.**

**• 6 Tier A/B rows from 5 source reports — the updated primary descriptive core, reported as row-level ranges without pooling. The pre-cutoff core contains five rows from four reports; the additional Fujii row is explicitly marked as a post-cutoff update.**

**• 51 AUROC estimates — the discrimination inventory, comprising 38 primary-designated estimates and 13 dependent or overlapping outputs; these are not 51 independent DTA rows.**

**• Dependence — Li 2025 contributes oral and stool rows, and Chen 2025b contributes two models from the same validation cohort. The updated nine-row set therefore represents seven source reports, not nine independent cohorts. Drop-both-Chen (7 rows/6 reports) and one-row-per-source-report (7/7) analyses assess sensitivity to duplicated report contributions. Original-cutoff analyses (5/4 descriptive; 8/6 exploratory) assess the temporal update separately. Residual cohort overlap cannot be fully modeled without participant-level covariance.**

This Supplementary Material contains five supplementary figure numbers (S1–S5), seven supplementary tables (S1A, S1B, and S2–S6), Supplementary Methods and interpretive qualifications (M1–M7), and descriptions of the audit workbook, reporting checklists, PROSPERO record, and OSF repository. No extra pooled estimate is reported for the Tier A/B descriptive core. The AUROC forests are incorporated as Supplementary Figure S2.

# **Supplementary Methods and interpretive qualifications**

## **M1. Search chronology and post hoc analytical hierarchy**

The original eight-database search ended on April 19, 2026. The September 3, 2026 PubMed/Europe PMC search was a targeted top-up covering subsequent records, not an update of all eight databases. The updated descriptive hierarchy is a post hoc revision-stage extension of the original evidence set. The word “primary” denotes the unpooled descriptive subset with more directly auditable cells; it does not mean that the updated six-row membership was prespecified in PROSPERO. Nor does “study” in the manuscript establish independent recruitment: source-report counts, model rows, sample-domain entries, and cohorts are distinct units.

The pre-cutoff Tier A/B core comprises Farrell 2012, Ren 2017, Li 2025 oral and stool, and Sammallahti 2025 (5 rows/4 reports; sensitivity 0.848–0.964; specificity 0.667–0.963). Adding Fujii 2026 yields the updated descriptive core (6/5), lowering the sensitivity range to 0.682–0.964 without changing the specificity range. Adding Chen 2025b models 1 and 2 and Lu 2019 produces the updated exploratory set (9/7). The corresponding pre-cutoff exploratory set is 8/6. Table S2 shows these roles side by side so that the targeted update cannot be mistaken for a complete database refresh. The updated set is the reference exploratory synthesis because it includes the additional eligible report available at revision, not because its fitted correlation is farther from a boundary. This post hoc reporting choice is accompanied by the original-cutoff temporal analyses and does not imply a complete database update.

Fujii 2026 analyses previously characterised linked cohorts and uses post-selection leave-one-out validation. It is one eligible report, not new independent recruitment or external validation. Its whole-PC row enters the updated descriptive and exploratory sets once; its two early-PC subgroup contrasts share participants and remain auxiliary fixed-threshold audit rows and dependent AUROC outputs. Earlier linked cohort reports are not counted as additional included studies merely because Fujii cites them; they must independently meet eligibility. The available source-level data cannot exclude all overlap among internationally reused cohorts.

## **M2. Executed statistical model, SROC, and uncertainty**

The reported quantitative results were executed in Python 3.12.13 with NumPy 2.3.5 and SciPy 1.17.0; figure rendering uses Matplotlib 3.10.8 and Pillow 12.3.0. The kit records input hashes, source code, execution output, fitted parameters, and numerical checks. Stata 19.5 scripts are supplied as companion replication code but were not executed for the reported results. The independent R package checks below are separate from the Python analysis used for all reported exploratory estimates.

The bivariate model uses binomial likelihoods for TP among cases and TN among controls with correlated normal random intercepts on logit sensitivity and logit specificity. Each model row is the fitting unit. Likelihoods were integrated by a two-dimensional product Gauss–Hermite rule with 25 nodes per dimension (625 pairs per row), optimized with SciPy L-BFGS-B, and checked with 35 nodes per dimension. The four reported fits differed by less than 0.00000006 in summary probabilities between the integration settings. Fixed-effect Wald intervals use the inverse observed Hessian estimated by central differences with a 0.001 step. Non-convergence or an invalid Hessian causes failure; reported values are not substituted from manuscript targets. Changing quadrature settings within the same implementation assesses numerical stability; it is not cross-software validation.

Figure 5 uses the updated nine-row model. The SROC is a reparameterization of that fitted bivariate model, not a second independently fitted model; the curve passes through its summary point. With β = log(σSp/σSe), the curve satisfies logit(Se) = λ exp(−β/2) + exp(−β) logit(1−Sp), with λ chosen from the two fitted mean logits. The 95% confidence contour uses the fixed-effect covariance on the two-logit scale and a χ² distribution with two degrees of freedom. The row-level prediction contour adds the estimated between-row covariance before transformation to ROC space. These approximate contours exclude uncertainty from same-report dependence, reconstructed cells, threshold selection, and model selection. They are not clinical performance guarantees.

The updated nine-row fit has random-effect correlation 0.867; the updated drop-both-Chen and one-row-per-report fits have correlations 0.776 and 0.781. None reaches the imposed bound. The original-cutoff eight-row fit reaches correlation tanh(3) = 0.995, so its covariance and uncertainty are particularly fragile. Figure 5 therefore shows only its summary as a temporal comparison. The number of source reports remains small for all models, including those without a boundary estimate.

Individual sensitivity and specificity intervals are Wilson intervals from the stated cells without continuity correction. Supplementary Figure S4 uses separate logit DerSimonian–Laird marginal models with 0.5 added to every 2×2 cell, including rows with no zero cell, to preserve the documented marginal-forest convention. That convention is not used in the binomial GLMM. τ² and I², where shown for these marginal models, are univariate summaries; a single bivariate I² is not asserted. The nine-row marginal logit τ² values are 0.06355 for sensitivity and 0.07925 for specificity. LR+, LR−, and DOR in Table S5 are transformations of unrounded operating points, not separately fitted outcomes.

An independent same-likelihood package check was executed in R 4.3.3 using GLMMadaptive 0.9-7. The same nine model rows from seven source reports were fitted as binomial successes/failures for sensitivity and specificity, with endpoint-specific fixed effects and correlated row-level random intercepts. Adaptive Gauss–Hermite integration used 15 nodes per dimension. The fit converged with a positive-definite observed Hessian and yielded sensitivity 0.857693 (95% CI 0.803015–0.899101) and specificity 0.803103 (0.728580–0.861066). All six summary accuracy estimates and interval limits agreed with the reference Python implementation after rounding to three decimals. This supports the reported summary accuracy calculations, not exact identity of every fitted parameter: random-effect correlations were 0.856 in the R check and 0.867 in Python. The kit includes unchanged inputs, scripts, versions, logs and comparisons.

A separate mature-package method-sensitivity check was executed in R 4.3.3 using mada 0.5.12 (reitsma; mvmeta 1.0.3) on the identical nine rows from seven reports. The no-covariate model used logit sensitivity and logit false-positive rate (alpha = 1), without continuity correction. Maximum likelihood converged and gave sensitivity 0.839602 (95% CI 0.788826–0.880026) and specificity 0.778185 (0.715011–0.830671); the default restricted maximum-likelihood fit also converged and gave 0.841738 (0.784700–0.885862) and 0.784115 (0.715875–0.839637), respectively. Package versions, unchanged input, executed script, console log, and estimates are supplied in the reproducibility kit. This is an independent method-sensitivity check using a normal–normal approximation to transformed proportions, not the binomial likelihood of the reported Python GLMM or its exact numerical replication. The R estimates are not substituted into the manuscript or figures.

The binomial GLMM remains the reference exploratory model because it models the stated 2×2 cell counts directly, avoiding the normal approximation for transformed proportions; this is relevant when some cells contain only one or two observations. All cells are positive, so no continuity correction was used in either bivariate approach. Differences of approximately 1.6–2.5 percentage points from mada reflect likelihood and estimation choices. On the common logit-sensitivity/logit-specificity scale, correlations were 0.921 (mada ML), 0.575 (mada REML), 0.867 (Python GLMM), and 0.856 (GLMMadaptive). This estimator sensitivity is consistent with weak determination of the covariance structure in this small, partly dependent dataset; confidence and prediction regions remain exploratory.

Deeks' test was prespecified solely as an exploratory assessment of small-study effects. With k < 10 and dependence between some rows, its output is not used to establish the absence of publication bias. Supplementary Figure S5 retains the original-cutoff eight-row/six-report analysis; the updated nine-row/seven-report output is separately identified in the kit.

## **M3. Dependence and deterministic sensitivity analyses**

The nine model rows represent seven source reports: Li contributes paired oral/stool rows, and Chen contributes two models from the same validation cohort. Their covariance is unavailable. The bivariate model therefore treats correlated rows as independent and may overstate precision or their contribution to the estimate. Removing both Chen rows leaves seven rows from six reports. A separate post hoc one-row-per-source-report check retains Farrell, Ren, Li oral, Chen model 1, Lu, Sammallahti, and Fujii (7/7). Selection used validation maturity, then reconstruction tier, then source order, not observed performance. Selecting a single row does not erase historical cohort reuse or small-sample uncertainty.

The row-level leave-one-out check of the unpooled six-row core is descriptive. Removing Fujii raises the minimum sensitivity from 0.682 to 0.848; thus, the endpoints of the range do depend on individual rows. The check means that no deletion changes the conclusion of a small, biased and heterogeneous evidence base, not that all rows produce interchangeable ranges. It does not estimate a pooled influence statistic or correct dependence between Li’s two rows.

## **M4. Clinical terminology and limits of interpretation**

Population screening means testing asymptomatic populations; early detection means identifying preclinical or early-stage disease before advanced presentation. High-risk surveillance is repeated assessment of selected high-risk individuals; diagnosis is evaluation prompted by symptoms or abnormal findings; adjunctive triage prioritizes imaging, EUS, or tissue confirmation. Literature screening is a separate review-process term. None of the included evidence establishes a validated stand-alone population-screening test, a demonstrated lead-time benefit, or improved patient outcomes.

Oral external-validation evidence is present in Nagata and Li but is variable: the inventoried external oral AUROCs include 0.520 and 0.502–0.575, respectively. Farrell’s final selected classifier is evaluated within the same cohort. Li’s two fixed-threshold core rows come from internal validation, while Sammallahti contributes an externally validated fixed-threshold core row. Stool has the clearest external discrimination programme, but Kartal and Nagata do not provide eligible fixed-threshold rows here. Validation maturity in Table S1A describes the best reported setting for the sample-domain entry, not necessarily the setting of every estimate in Tables S4 and S6.

Clinical maturity, feasibility, and utility should be read separately. A numerical difference between oral and stool rows is not a head-to-head comparison. Multi-taxon panels, serial sampling, and integration with CA19-9 or other modalities are design proposals whose reproducibility, calibration, net benefit, and clinical impact remain to be tested. Biological associations do not establish causation. Future comparator designs should include benign pancreatic mimics as the primary clinical contrast and may retain healthy controls secondarily. Statements about panels or serial sampling do not establish their superiority or reliability in routine care.

## **M5. Comparison with the oral-microbiome DTA review**

Koopaie et al. (2025; doi: 10.1080/20002297.2025.2571188) reviewed nine studies comprising 188 study units and 6,601 participants. Their single-oral-microbe synthesis reported sensitivity 0.65, specificity 0.70, and DOR 4.85. The multiple-oral-microbe subgroup reported sensitivity 0.81 (95% CI 0.75–0.86) and specificity 0.78 (0.74–0.81). Our 0.86/0.80 exploratory operating point combines oral, stool, and tongue-coat classifier rows under a different reconstruction and validation hierarchy; it cannot be interpreted as outperforming their oral-only synthesis. Taxon-level study units, classifier-level rows, controls, thresholds, and validation settings differ. Both reviews support diagnostic promise with substantial restrictions on clinical interpretation.

Koopaie was incorporated during the revision-stage contextual reference audit and is explicitly discussed here; it was not a newly eligible primary DTA report from the post-cutoff top-up. Its 2025 publication predates April 19, 2026. The available search records do not establish why it was absent from the earlier narrative bibliography. This contextual addition does not change the PRISMA included-report count or supply any 2×2 row.

## **M6. AUROC provenance and validation-maturity reconciliation**

The 51-estimate inventory is 43 legacy estimates plus five additional estimates recovered from already included full texts plus three Fujii outputs. The five recoveries are Nagata oral external 0.520; Half integrated Israeli 0.833 and integrated Ren 0.798; Sammallahti genus 0.870 and phylum 0.850. The three Fujii estimates are 0.804, 0.780, and 0.739. This is 48 pre-cutoff plus three post-cutoff estimates, designated as 38 primary outputs and 13 dependent or overlapping outputs. “Primary” in this AUROC inventory is an output-selection label, not membership in the fixed-threshold core. Recovery of AUROCs did not create additional fixed-threshold cells.

Validation maturity across the 21 sample-domain entries is 6 external, 8 internal hold-out, 4 internal resampling, and 3 without independent validation. Relative to the former 7/6/3/4 distribution, Farrell was reclassified as same-cohort rather than external; the two Tavanaeian domain entries as internal hold-out rather than none; and the newly included Fujii entry as internal resampling. Tavanaeian stool remains in the descriptive map and AUROC inventory but is omitted from the 20-record plotted risk-of-bias set because the reported sensitivity, specificity and accuracy fields did not yield a stable model-based row. This accounting does not validate its apparently perfect AUROC.

## **M7. Author verification and repository transparency**

The available package documents structured extraction, source-location auditing, computational checking, and revision-stage reconciliation. Reviewer-level records sufficient to demonstrate independent duplicate screening, extraction, or risk-of-bias adjudication were not available for verification. Such independent duplicate procedures are therefore not claimed. The targeted top-up sheet records search concepts, aggregate yields, and three detailed decisions, but it does not enumerate every one of the 19 screened records. This remains a review-process reporting limitation until a complete source-based screening log is provided; aggregate arithmetic is not a substitute for that log.

The available records do not establish completed independent author verification of eligibility decisions, source cells, risk-of-bias judgments, references, code outputs, or interpretations. The named authors retain responsibility for these checks and the final content. A complete line-by-line log of the 19-record targeted top-up is unavailable.

The data repository is https://osf.io/h358f/ (DOI: 10.17605/OSF.IO/H358F). The submitted-version files are publicly accessible without login. Proof-stage corrections are provided separately.

# **Supplementary Figures**

**Supplementary Figure S1. Distribution of included entries by publication year and sample domain.**

This figure displays 21 sample-domain entries from 16 included reports/publications by publication year and sample domain. Entries are not independent-cohort counts.

***Figure S1. Distribution of 21 sample-domain entries from 16 included reports/publications by publication year and sample domain. Stool entries dominate the later literature; oral/saliva entries span the full period; tongue coat is represented by one entry.***

**Supplementary Figure S2. AUROC discrimination inventory (oral/tongue coat and stool domains).**

This two-panel figure presents the 51-estimate AUROC inventory: 38 primary-designated estimates and 13 dependent or overlapping outputs. Panel A contains n = 14 oral/saliva or tongue-coat estimates (11 primary and 3 dependent), and Panel B contains n = 37 stool estimates (27 primary and 10 dependent). Filled coloured markers indicate primary estimates with an available 95% confidence interval, open coloured markers indicate primary estimates without an available interval, and grey open squares indicate dependent or overlapping outputs. Lu 2019 is displayed with its reported 95% confidence interval (0.697–0.891). These are discrimination estimates across thresholds, not independent fixed-cutoff DTA rows.

*Panel A. Oral/saliva and tongue coat (n = 14 estimates).*

*Panel B. Stool (n = 37 estimates).*

***Figure S2. AUROC discrimination inventory. Panel A: n = 14 oral/saliva and tongue-coat estimates. Panel B: n = 37 stool estimates. Reference lines are shown at 0.5, 0.7, and 0.9. Lu 2019 is shown with its reported 95% confidence interval (0.697–0.891). This descriptive inventory includes reports both with and without reconstructable fixed-threshold data; AUROCs were not entered as fixed-threshold observations.***

**Supplementary Figure S3. Individual fixed-threshold estimates by sample domain (no pooling).**

This descriptive plot displays all nine fixed-threshold model rows from seven source reports as individual sensitivity and specificity estimates with 95% Wilson confidence intervals. Fujii 2026 is visually identified as the post-cutoff targeted-top-up row. Each row’s sample domain is identified; no within-domain or between-domain pooling is performed.

***Figure S3. Individual fixed-threshold estimates with sample domain identified. No subgroup-effect inference or pooled domain estimate is reported.***

**Supplementary Figure S4. Univariate marginal sensitivity and specificity forests for the updated nine-row exploratory set.**

The two panels display separate univariate DerSimonian–Laird random-effects summaries of the nine fixed-threshold rows from seven source reports used in the updated exploratory bivariate model. Sensitivity was 0.83 (95% CI 0.77–0.88) and specificity was 0.77 (0.71–0.83). These marginal estimates are supporting forest-plot visualizations only; the reference exploratory synthesis is the nine-row bivariate result reported in the main text. An all-cell 0.5 correction was used for the logit-based marginal models; it was not applied to the binomial-likelihood model or Wilson intervals (M2).

*Panel A. Marginal sensitivity.*

*Panel B. Marginal specificity.*

***Figure S4. Separate univariate DerSimonian–Laird marginal forests for nine model rows from seven source reports. Fujii 2026 is identified as a post-cutoff update. These supporting summaries are not the reference exploratory operating point.***

**Supplementary Figure S5. Deeks funnel plot for small-study effects (exploratory).**

Deeks’ funnel-plot output is retained as an ancillary original-cutoff display. It contains eight pre-cutoff rows from six source reports, includes approximate Tier C rows, and is underpowered; the regression coefficient was 17.08 (p = 0.346). Its denominator differs deliberately from the updated nine-row model. It is not interpreted as evidence for or against publication bias. The manuscript's k < 10 non-interpretation rule applies; dependence further reduces the effective number of independent observations.

***Figure S5. Deeks’ funnel plot for the original-cutoff set (exploratory and non-interpretable for publication-bias inference; eight rows from six source reports; coefficient 17.08, p = 0.346).***

# **Supplementary Tables**

**Table S1A. Stratified evidence map—comparator structure and validation maturity for the 21 sample-domain entries.**

| **Source report** | **Domain** | **Primary comparator** | **Benign-mimic support** | **Validation maturity** | **External validation** |
| --- | --- | --- | --- | --- | --- |
| Farrell 2012 | oral | healthy only | direct secondary | no independent validation | candidate markers only; final model no |
| Nagata 2022 | oral | mixed nonpancreatic controls | none | external validation | yes |
| Chen 2023 | oral | healthy only | contextual only | internal holdout | no |
| Li 2024 | oral | benign pancreatic only | primary direct | internal resampling only | no |
| Li(Pengyu)2025 / Cancer Letters | oral | healthy only | none | external validation | yes |
| Tavanaeian 2025 | oral | healthy only | none | internal hold-out | no |
| Ren 2017 | stool | healthy only | none | no independent validation | no |
| Half 2019 | stool | mixed nonpancreatic controls | contextual only | internal holdout | no |
| Zhou 2021 | stool | healthy only | direct secondary | internal holdout | no |
| Kartal 2022 | stool | mixed nonpancreatic controls | direct secondary | external validation | yes |
| Nagata 2022 | stool | mixed nonpancreatic controls | direct secondary | external validation | yes |
| Chen 2023 | stool | healthy only | contextual only | internal holdout | no |
| Yang 2023 | stool | healthy only | none | no independent validation | no |
| Li 2024 | stool | benign pancreatic only | primary direct | internal resampling only | no |
| Chen 2025b | stool | healthy only | none | internal holdout | no |
| Li(Pengyu)2025 / Cancer Letters | stool | healthy only | none | external validation | yes |
| Villani 2025 | stool | healthy only | none | internal holdout | no |
| Lu 2019 | tongue coat | healthy only | other nonpancreatic only | internal resampling only | no |
| Sammallahti 2025 | stool | healthy only | none | external validation | yes |
| Tavanaeian 2025† | stool | healthy only | none | internal hold-out | no |
| Fujii 2026 | stool | healthy only | direct secondary high-risk | internal resampling only | no |

*Note. Counts refer to 21 sample-domain entries, not independent reports or cohorts. Healthy-control primary comparisons accounted for 15/21 entries, benign pancreatic comparators for 2/21, and mixed non-pancreatic controls for 4/21. Validation maturity was external in 6 entries, internal hold-out in 8, internal resampling in 4, and absent in 3. The 20-record plotted risk-of-bias set contains all entries except the Tavanaeian 2025 stool marker (†), whose published performance fields were internally inconsistent; this exclusion does not remove it from the descriptive evidence map.*

Table S1B. Source-report- and sample-domain-level microbiome transportability extraction.

| **Source report / domain** | **Sampling, treatment, and medication context** | **Storage / DNA extraction** | **Assay / platform** | **Contamination, negative-control, and batch reporting** |
| --- | --- | --- | --- | --- |
| Farrell 2012 Oral; USA | Prospectively banked saliva; treatment-naive; antibiotic/PPI NR | −80°C; MO BIO UltraClean kit | HOMIM 16S microarray + qPCR | Negative controls, contamination control, and batch correction NR |
| Nagata 2022 Oral; JP/ES/DE | Pretreatment morning saliva before breakfast; PPI recorded; antibiotic exclusion NR | RNALater → 4°C (12 h) → −20°C (24 h) → −80°C; extraction per report | Shotgun metagenomics; ancillary 16S V3–V4 | Batch handling reported; negative/contamination controls NR |
| Chen 2023 Oral; China | Prospective pretreatment saliva; antibiotic exclusion and PPI recorded | Immediate −80°C; QIAamp DNA Mini kit | 16S V3–V4; Illumina platform not fully specified | Negative controls, contamination control, and batch correction NR |
| Li 2024 Oral; China | Pretreatment tongue/oral sample; antibiotic exclusion reported; PPI NR | −80°C within 1 h; E.Z.N.A. kit | 16S V3–V4; NovaSeq PE250 | Negative controls, contamination control, and batch correction NR |
| Li (Pengyu) 2025 Oral; China + external sets | Pretreatment; antibiotic exclusion reported; PPI NR | −80°C within 1 h; extraction per prior methods | 16S amplicon; Illumina NovaSeq | Negative controls, contamination control, and batch correction NR |
| Tavanaeian 2025 Oral; Iran | Saliva from cases/controls; treatment and antibiotic/PPI handling NR | Extracted DNA stored at −20°C; saliva kit reported | Targeted 16S qPCR; LightCycler 96 | Negative controls, contamination control, and batch correction NR |
| Ren 2017 Stool; China | Immediate aliquoting; treatment-naive; antibiotic exclusion reported; PPI NR | Immediate −80°C; >2 h at room temperature rejected; phenol/chloroform + bead beating/Qiagen purification | 16S V3–V4; MiSeq | Sequencing depth/database reported; negative/contamination and batch controls NR |
| Half 2019 Stool; Israel | Sampled near diagnosis before treatment; antibiotic exclusion reported; PPI NR | −80°C; MO BIO PowerSoil kit | 16S V3–V4; Illumina MiSeq | Negative controls, contamination control, and batch correction NR |
| Zhou 2021 Stool; China | Fresh stool transported within 2 h; antibiotic exclusion reported; treatment/PPI NR | Below −80°C; QIAamp PowerFecal Pro kit | Shotgun metagenomics; Illumina HiSeq | Sequencing depth reported; negative/contamination and batch controls NR |
| Kartal 2022 Stool; Spain/Germany | At recruitment; treatment-naive; antibiotic/PPI handling NR | RNALater → 4°C → −20°C → −80°C; AllPrep PowerFecal DNA/RNA kit | Shotgun metagenomics + ancillary 16S V4 | Negative and contamination controls reported; batch correction NR |
| Nagata 2022 Stool; JP/ES/DE | Pretreatment stool; treatment-naive; PPI recorded; antibiotic exclusion NR | RNALater → 4°C (12 h) → −20°C (24 h) → −80°C; extraction per report | Shotgun metagenomics; ancillary 16S V3–V4 | Batch handling reported; negative/contamination controls NR |
| Chen 2023 Stool; China | Prospective pretreatment stool; antibiotic exclusion and PPI recorded | Immediate −80°C; QIAamp Fast DNA Stool Mini kit | 16S V3–V4; Illumina platform not fully specified | Negative controls, contamination control, and batch correction NR |
| Yang 2023 Stool; China | Untreated cases; antibiotic exclusion reported; PPI NR | Immediate −80°C; Omega Mag-Bind Soil kit | 16S V3–V4; Illumina | Sequencing depth/database reported; negative/contamination and batch controls NR |
| Li 2024 Stool; China | Pretreatment stool; antibiotic exclusion reported; PPI NR | −80°C within 1 h; E.Z.N.A. Stool DNA kit | 16S V3–V4; NovaSeq PE250 | Negative controls, contamination control, and batch correction NR |
| Chen 2025b Stool; China | Pretreatment; antibiotic exclusion reported; PPI NR | Sterile tubes with 2% glycerol; −80°C within 1 h; extraction kit/vendor NR | Shotgun metagenomics; NovaSeq X Plus | Negative controls, contamination control, and batch correction NR |
| Li (Pengyu) 2025 Stool; China + external sets | Pretreatment; antibiotic exclusion reported; PPI NR | −80°C within 1 h; extraction per prior methods | 16S amplicon; Illumina NovaSeq | Negative controls, contamination control, and batch correction NR |
| Villani 2025 Stool; Italy | Cases/controls; treatment-naive; antibiotic exclusion reported; PPI NR | −80°C; QIAamp Fast DNA Stool Mini kit | 16S V3–V4; Illumina MiSeq 2 × 300 | Database reported; negative/contamination and batch controls NR |
| Lu 2019 Tongue coat; China | Tongue coat from stage-I PHC/controls; treatment-naive; antibiotic exclusion reported; PPI NR | Storage NR; Qiagen Mini kit | 16S V3–V4; Illumina MiSeq | Database reported; negative/contamination and batch controls NR |
| Sammallahti 2025 Stool; Finland/Iran | Iranian cohort sampled before treatment; Finnish preoperative cohort included some post-stent/neoadjuvant cases; antibiotic exclusion reported | Cohort-specific protocols; Finnish stabilizer tubes; extraction protocol reported | 16S V4; Ion Torrent PGM | Sequencing depth/database reported; contamination and batch controls NR |
| Tavanaeian 2025† Stool; Iran | Stool from cases/controls; treatment and antibiotic/PPI handling NR | Raw-stool storage NR; QIAamp Stool kit after lysozyme pretreatment | Targeted 16S qPCR; LightCycler 96 | Negative controls, contamination control, and batch correction NR; performance table internally inconsistent |
| Fujii 2026 Stool; Japan | Previously banked stool; treatment and antibiotic/PPI handling NR in the report | Storage described in linked cohort reports; extraction described in publisher methods | Targeted fecal qPCR | Negative controls, contamination control, batch handling, and data/code availability NR in the primary report |

Note. NR means not reported or not recoverable from the available report; it does not mean that a procedure or exposure was absent. These fields are descriptive and were not used in meta-regression. Rows correspond to 21 sample-domain entries, not independent cohorts. †The Tavanaeian 2025 stool entry is retained descriptively but excluded from the 20-record plotted risk-of-bias set because its published performance fields were internally inconsistent.

Table S2. Analytical hierarchy and interpretation of all reported summary outputs.

| **Analysis** | **Rows / source reports** | **Composition** | **Model / display** | **Result** | **Interpretive role** |
| --- | --- | --- | --- | --- | --- |
| Updated primary Tier A/B core | 6 / 5 | Farrell; Ren; Li oral and stool; Sammallahti; Fujii | No pooling; Wilson CIs | Se 0.682–0.964; Sp 0.667–0.963 | Primary descriptive subset; post hoc update |
| Updated expanded set | 9 / 7 | Updated core + Chen M1/M2 + Lu | Bivariate binomial-logit GLMM; SROC with confidence and prediction regions | Se 0.86 (0.80–0.90); Sp 0.80 (0.73–0.86) | Reference exploratory synthesis |
| Updated marginal forests | 9 / 7 | Same nine rows | Separate univariate DerSimonian–Laird models | Se 0.83 (0.77–0.88); Sp 0.77 (0.71–0.83) | Supporting visualization only |
| Drop-both-Chen, updated set | 7 / 6 | Updated set excluding both Chen rows | Bivariate binomial-logit GLMM | Se 0.86 (0.80–0.91); Sp 0.80 (0.71–0.87) | Dependency sensitivity |
| One row per source report, updated set | 7 / 7 | Farrell; Ren; Li oral; Chen M1; Lu; Sammallahti; Fujii | Post hoc deterministic bivariate model | Se 0.86 (0.79–0.91); Sp 0.82 (0.72–0.88) | Dependency sensitivity |
| Pre-cutoff Tier A/B subset | 5 / 4 | Updated core excluding Fujii | No pooling; Wilson CIs | Se 0.848–0.964; Sp 0.667–0.963 | Temporal robustness; original cutoff |
| Original-cutoff expanded set | 8 / 6 | Updated expanded set excluding Fujii | Bivariate binomial-logit GLMM | Se 0.87 (0.82–0.91); Sp 0.82 (0.74–0.88) | Temporal robustness; correlation boundary |
| Deeks, original cutoff | 8 / 6 | Original-cutoff expanded set | Exploratory weighted regression | Coefficient 17.08; p = 0.346 | Ancillary only; no publication-bias inference |

**Table S3. Search yields and study-selection flow.**

| **Study-selection / evidence-structure item** | **Count or disposition** |
| --- | --- |
| Original records identified across eight databases | 1,074 |
| Original-search duplicates removed | 492 |
| Original records screened (title/abstract) | 582 |
| Original records excluded at title/abstract | 540 |
| Original reports sought and retrieved | 42 |
| Original reports assessed for eligibility | 42 |
| Original full-text exclusions: no DTA-ready model/extractable accuracy | 13 |
| Original full-text exclusions: out-of-scope biomarker/sample domain/mixed design | 10 |
| Original full-text exclusions: secondary re-analysis/overlapping cohort/target mismatch | 4 |
| Targeted top-up (PubMed + Europe PMC; 20 Apr–3 Sep 2026) | 35 source records → remove 15 cross-source duplicates + 1 legacy-library record → 19 new reports screened → 16 title/abstract exclusions → 3 detailed checks → 2 exclusions → 1 eligible report |
| Detailed top-up exclusion: Kharazishvili et al. 2026 | Microbiome component reported no eligible AUROC/sensitivity/specificity; the reported AUROC concerned leukocyte telomere length |
| Detailed top-up exclusion: Tavanaeian machine-learning companion (2026) | Overlapping 20/20 cohort already represented; no new independent report-level diagnostic evidence |
| Eligible top-up report | Fujii 2026; one report and one stool sample-domain entry |
| Included reports/publications (updated PRISMA count) | 16 |
| Sample-domain entries | 21 |
| Model-based plotted risk-of-bias records | 20 |
| AUROC estimates inventoried | 51 |
| Fixed-threshold model rows | 9 |
| Updated Tier A/B core rows / source reports | 6 / 5 (pre-cutoff subset: 5 / 4) |
| Original-cutoff expanded model rows / source reports | 8 / 6 |
| Updated exploratory model rows / source reports | 9 / 7 (includes one post-cutoff Fujii row) |

*Note. The original search covered eight databases through April 19, 2026. The September 3 targeted top-up was restricted to PubMed and Europe PMC; it did not rerun all eight databases. Of 35 source records (16 and 19, respectively), 15 cross-source duplicates and one legacy-library record were removed. Nineteen new records were screened: 16 were excluded at title/abstract, three were checked in detail, two were excluded, and Fujii 2026 was included. The audit workbook provides search concepts, strings, aggregate yields and three detailed decisions, but not a complete line-by-line log of all 19 records. A separate publisher-directed check found no further eligible report. The updated total is 16 included reports/publications.*

**Table S4. Fixed-threshold 2×2 reconstruction and candidate-row audit log.**

| **Source report** | **Domain** | **Reported metric** | **Source location** | **Cases / Controls** | **TP/FP/TN/FN Status / role** |
| --- | --- | --- | --- | --- | --- |
| Farrell 2012 | oral | se_sp_n | Abstract + validation cohort description | 28 / 28 | 27/5/23/1 Final Tier A core |
| Li(Pengyu)2025 / Cancer Letters | oral | se_sp_auc_n | Results/Figure 6 | 29 / 27 | 27/1/26/2 Final Tier B core |
| Tavanaeian 2025 | oral | screening_summary | screening workbook candidate sheet | 20 / 20 | AUROC-only AUROC only; not in DTA |
| Ren 2017 | stool | se_sp_auc_n | Abstract + Figure 7 | 85 / 57 | 73/19/38/12 Final Tier A core |
| Chen 2025b | stool | se_sp_auc_n | Validation results section | 20 / 20 | 17/3/17/3 Final Tier C-M1 |
| Li(Pengyu)2025 / Cancer Letters | stool | se_sp_auc_n | Results/Figure 6 | 29 / 27 | 26/7/20/3 Final Tier B core |
| Villani 2025 | stool | se_sp_auc_n | Figure 3C / results text | NR / NR | Not reconstructable / Superseded; no fixed-threshold 2×2 |
| Lu 2019 | tongue coat | se_sp_auc_n | Main text (PHC vs HC ROC paragraph) | 30 / 25 | 23/5/20/7 Final Tier C |
| Farrell 2012 | oral | se_sp_n | Full text results / Figure 3B | 28 / 27 | 24/12/15/4 Audit only; alternate Farrell model |
| Li(Pengyu)2025 / Cancer Letters | oral | se_sp_auc_n | Results/Figure 6B1-B2 | 29 / 27 | 27/1/26/2 Audit only; duplicate source record |
| Chen 2025b | stool | se_sp_auc_n | Methods 6:4 split + validation results | 20 / 20 | 16/4/16/4 Final Tier C-M2 |
| Li(Pengyu)2025 / Cancer Letters | stool | se_sp_auc_n | Results/Figure 6D1-D2 | 29 / 27 | 26/7/20/3 Audit only; duplicate source record |
| Villani 2025 | stool | se_sp_auc_n | Full text Figure 3C / results | NR / NR | Not reconstructable / Superseded; no fixed-threshold 2×2 |
| Lu 2019 | tongue coat | se_sp_n | Body text around Figure 5(c) and Table 1 | 30 / 25 | 23/5/20/7 Audit only; duplicate source record |
| Chen 2025b | stool | se_sp_auc_n | Results section / Figure 2E-F description | / | AUROC-only AUROC only; not in DTA |
| Chen 2025b | stool | se_sp_auc_n | Results section / Figure 3A description | / | AUROC-only AUROC only; not in DTA |
| Chen 2025b | stool | se_sp_auc_n | Results section / Figure 3A description | / | AUROC-only AUROC only; not in DTA |
| Sammallahti 2025 | stool | se_sp_auc_n | Abstract / Results / classifier performance table in full text | 33 / 35 | 28/4/31/5 Final Tier A core |
| Fujii 2026 | stool | se_sp_auc_n | OUP full text, model-performance results | 22 / 65 | 15/18/47/7 Updated Tier A core; post-cutoff targeted top-up; cutoff 0.2818 |
| Half 2019 | stool | se_sp_auc_n | Held-out validation results; exact subset composition not reported | 13 / 5 (conditionally recovered; not reported) | 10/1/4/3 Auxiliary audit row only; excluded because the 70/30 validation subset denominator and case/control composition were not explicitly reported |
| Sammallahti 2025 [phylum] | stool | se_sp_auc_n | Results Table 5 | 33 / 35 | 30/5/30/3 / Correlated auxiliary Tier A; excluded from formal models |
| Sammallahti 2025 [genus] | stool | se_sp_auc_n | Results Table 5 | 33 / 35 | 26/7/28/7 / Correlated auxiliary Tier A; excluded from formal models |
| Fujii 2026 [early PC vs HC] | stool | se_sp_auc_n | OUP full text, Table 3 | 17 / 65 | 12/15/50/5 / Overlapping auxiliary Tier A; cutoff 0.2818 |
| Fujii 2026 [early PC vs high-risk] | stool | se_sp_auc_n | OUP full text, Table 3 | 17 / 64 | 12/24/40/5 / Overlapping auxiliary Tier A; cutoff 0.2818 |

Note. TP/FP/TN/FN are the four-cell values used for DTA analysis. A/B/C are review-specific data-reconstruction verification tiers, not evidence-quality grades: Tier A denotes directly reported or exactly recoverable integer cells, Tier B near-integer recovery from a defined validation sample with consistency checks, and Tier C approximate reconstruction used only in exploratory analyses. These tiers were not prespecified in PROSPERO and do not replace QUADAS-2, PROBAST, or the adapted, GRADE-informed certainty appraisal. Wilson intervals do not include reconstruction uncertainty. Six Tier A/B rows form the updated unpooled primary core; the pre-cutoff subset is five rows/four reports. Three Tier C rows enter the updated nine-row/seven-report exploratory synthesis. Fujii is the one post-cutoff Tier A row, not an independent external-validation cohort. Audit-only, duplicate-source, and AUROC-only rows were not entered. Five auxiliary audit rows remain excluded. The prior Villani reconstruction is superseded, and Half 2019 remains auxiliary because the exact case/control composition of its internal-validation subset was not reported. For Li 2025, 29 cases and 27 controls are approximate counts inferred from the 7:3 split; the source flow diagram reports 57 validation participants without a case/control breakdown. The 29/27 counts are proxies, not reported validation counts.

**Table S5. Sensitivity-analysis summaries and dependency checks.**

| **Analysis layer** | **Rows / source reports** | **Sensitivity (95% CI)** | **Specificity (95% CI)** | **Interpretation** | **LR+ / LR− / DOR** |
| --- | --- | --- | --- | --- | --- |
| Updated exploratory bivariate model | 9 / 7 | 0.86 (0.80–0.90) | 0.80 (0.73–0.86) | Reference exploratory synthesis | 4.36 / 0.18 / 24.58 |
| Updated univariate marginals | 9 / 7 | 0.83 (0.77–0.88) | 0.77 (0.71–0.83) | Supporting visualization only | 3.68 / 0.22 / 16.74 |
| Drop-both-Chen, updated set | 7 / 6 | 0.86 (0.80–0.91) | 0.80 (0.71–0.87) | Dependency sensitivity | 4.37 / 0.17 / 25.86 |
| One row per report, updated set | 7 / 7 | 0.86 (0.79–0.91) | 0.82 (0.72–0.88) | Post hoc dependency sensitivity | 4.69 / 0.17 / 27.30 |
| Updated Tier A/B core | 6 / 5 | 0.682–0.964 | 0.667–0.963 | Unpooled descriptive range | Not pooled / N/A |
| Pre-cutoff Tier A/B subset | 5 / 4 | 0.848–0.964 | 0.667–0.963 | Unpooled temporal robustness | Not pooled / N/A |
| Original-cutoff bivariate model | 8 / 6 | 0.87 (0.82–0.91) | 0.82 (0.74–0.88) | Temporal robustness; correlation boundary | 4.76 / 0.16 / 29.58 |
| Deeks, original cutoff | 8 / 6 | N/A | N/A | Coefficient 17.08; p = 0.346; non-interpretable | N/A |

*Note. The updated nine-row/seven-report bivariate model yielded sensitivity 0.86 (95% CI 0.80–0.90) and specificity 0.80 (0.73–0.86). Excluding both Chen rows yielded 0.86 (0.80–0.91) and 0.80 (0.71–0.87), while retaining one row per source report yielded 0.86 (0.79–0.91) and 0.82 (0.72–0.88). These three updated fits did not reach the random-effect-correlation bound. Removing Fujii for the original-cutoff analysis yielded 0.87 (0.82–0.91) and 0.82 (0.74–0.88); its correlation reached the numerical bound. The six-row/five-report updated core and five-row/four-report pre-cutoff core were not pooled. LR+, LR−, and DOR are algebraic transformations of the corresponding unrounded summary estimates and have no separately estimated confidence intervals. All pooled results remain exploratory; neither temporal nor dependency sensitivity analyses establish independent validation.*

**Table S6. AUROC discrimination inventory (51 estimates).**

| **Source report** | **Domain** | **Model** | **AUROC** | **95% CI** | **Evaluation setting** |
| --- | --- | --- | --- | --- | --- |
| Li(Pengyu)2025 / Cancer Letters | oral | Oral microbiome + CA19-9 classifier | 1.000 | 1.000-1.000 | hybrid model |
| Tavanaeian 2025 | oral | Salivary N. elongata qPCR marker | 0.970 | 0.900–1.000‡ | internal hold-out |
| Li 2024 | oral | Oral random forest classifier (LOOCV) | 0.963 | 0.934-0.991 | whole cohort |
| Li(Pengyu)2025 / Cancer Letters | oral | Oral random forest classifier (internal validation) | 0.963 | 0.922-1.000 | internal validation |
| Li 2024 | oral | Oral RF classifier + CA19-9 | 0.963 | 0.935-0.991 | hybrid model |
| Chen 2023 | oral | 10-genus oral RF panel | 0.916 | 0.832-1.000 | discovery |
| Farrell 2012 | oral | N. elongata + S. mitis qPCR panel | 0.900 | 0.780-0.960 | same-cohort final model |
| Nagata 2022 | oral | Oral species-level random forest classifier | 0.820 | NR | whole cohort |
| Farrell 2012 | oral | G. adiacens + S. mitis qPCR panel | 0.700 | 0.560-0.810 | same-cohort final model |
| Li(Pengyu)2025 / Cancer Letters | oral | Oral RF classifier - external Spanish cohort | 0.575 | NR | external validation |
| Nagata 2022 | oral | Oral species-level RF model - Spanish external validation | 0.520 | NR | external; dependent |
| Li(Pengyu)2025 / Cancer Letters | oral | Oral RF classifier - external Chinese cohort | 0.505 | NR | external validation |
| Li(Pengyu)2025 / Cancer Letters | oral | Oral RF classifier - external Japanese cohort | 0.502 | NR | external validation |
| Villani 2025 | stool | Whole-cohort random forest classifier | 1.000 | 1.000-1.000 | whole cohort |
| Tavanaeian 2025 | stool | Fecal R. intestinalis qPCR marker | 1.000 | 1.000-1.000 | internal hold-out |
| Li(Pengyu)2025 / Cancer Letters | stool | Fecal microbiome + CA19-9 classifier | 0.998 | 0.992-1.000 | hybrid model |
| Chen 2025b | stool | Model-1 genus signature + CA19-9 | 0.977 | 0.920-0.992 | hybrid model |
| Chen 2025b | stool | Model-2 species signature + CA19-9 | 0.953 | 0.905-0.978 | hybrid model |
| Yang 2023 | stool | Streptococcus1 marker ROC | 0.927 | 0.872–0.982§ | whole cohort |
| Chen 2025b | stool | Model-1 genus-level validation classifier | 0.923 | 0.893-0.952 | internal validation |
| Li 2024 | stool | Fecal RF classifier + CA19-9 | 0.909 | 0.851-0.968 | hybrid model |
| Zhou 2021 | stool | Fecal microbial biomarker combination | 0.907 | 0.774-1.000 | internal test set (30% holdout) |
| Li 2024 | stool | Fecal random forest classifier (LOOCV) | 0.894 | 0.835-0.954 | whole cohort |
| Li(Pengyu)2025 / Cancer Letters | stool | Fecal random forest classifier (internal validation) | 0.890 | 0.805-0.975 | internal validation |
| Zhou 2021 | stool | Fecal microbial biomarker combination | 0.889 | 0.735-1.000 | internal test set (30% holdout) |
| Sammallahti 2025 | stool | Selected family-level logistic regression classifier | 0.880 | 0.780-0.970 | external validation |
| Li(Pengyu)2025 / Cancer Letters | stool | Fecal RF classifier - external Chinese cohort | 0.878 | 0.809-0.948 | external validation |
| Sammallahti 2025 [genus] | stool | Genus-level SVM classifier, selected variables | 0.870 | 0.780-0.950 | external; dependent |
| Chen 2023 | stool | 10-genus fecal RF panel | 0.856 | 0.740-0.972 | discovery |
| Chen 2025b | stool | Model-2 species-level validation classifier | 0.853 | 0.801-0.905 | internal validation |
| Sammallahti 2025 [phylum] | stool | Phylum-level LR classifier, total variables | 0.850 | 0.740-0.950 | external; dependent |
| Kartal 2022 | stool | Model-2 PDAC-enriched fecal classifier (German validation) | 0.850 | NR | external validation |
| Ren 2017 | stool | 40-genera fecal classifier | 0.842 | 0.777-0.907 | whole cohort |
| Kartal 2022 | stool | Model-1 27-species fecal metagenomic classifier | 0.840 | NR | discovery |
| Half 2019 | stool | Integrated RF model on Israeli validation data | 0.833 | 0.616-1.000 | within-cohort; dependent |
| Nagata 2022 | stool | Gut species model applied to German cohort | 0.830 | NR | external validation |
| Kartal 2022 | stool | Model-1 27-species fecal classifier (German validation) | 0.830 | NR | external validation |
| Half 2019 | stool | Random forest validation model | 0.825 | 0.636-1.000 | internal validation |
| Half 2019 | stool | Integrated RF model on Ren validation data | 0.798 | 0.682-0.914 | within-cohort; dependent |
| Nagata 2022 | stool | Gut species-level random forest classifier | 0.780 | NR | whole cohort |
| Kartal 2022 | stool | Fecal metagenomic model vs CP | 0.750 | NR | discovery |
| Nagata 2022 | stool | Gut species model applied to Spanish cohort | 0.740 | NR | external validation |
| Kartal 2022 | stool | Model-2 PDAC-enriched fecal classifier | 0.710 | NR | discovery |
| Nagata 2022 | stool | Gut species-level RF model vs IPMN | 0.700 | NR | whole cohort |
| Nagata 2022 | stool | Gut species-level RF model vs CP | 0.680 | NR | whole cohort |
| Li(Pengyu)2025 / Cancer Letters | stool | Fecal RF classifier - external Japanese cohort | 0.649 | 0.566-0.733 | external validation |
| Li(Pengyu)2025 / Cancer Letters | stool | Fecal RF classifier - external Spanish cohort | 0.501 | 0.375-0.626 | external validation |
| Fujii 2026 [early PC vs HC] | stool | PC probability score, early-PC subgroup vs healthy controls | 0.804 | 0.692-0.899 | post-cutoff; dependent / overlapping; cutoff 0.2818 |
| Fujii 2026 | stool | Three-marker PC probability score (post-selection LOOCV) | 0.780 | 0.671-0.877 | post-cutoff; post-selection LOOCV; cutoff 0.2818 |
| Fujii 2026 [early PC vs high-risk] | stool | PC probability score, early-PC subgroup vs pancreatic high-risk group | 0.739 | 0.617-0.849 | post-cutoff; dependent / overlapping; cutoff 0.2818 |
| Lu 2019 | tongue coat | 4-genera tongue-coating ROC model | 0.802 | 0.697-0.891 | whole cohort |

*Note. The 51 AUROC estimates comprise 38 primary-designated estimates and 13 dependent or overlapping outputs across 21 sample-domain entries; they are not 51 independent DTA rows. Relative to the Round3 43-estimate inventory, five pre-existing full-text estimates were recovered (Nagata oral external, 0.520; Half integrated Israeli, 0.833; Half integrated Ren, 0.798; Sammallahti genus, 0.870; Sammallahti phylum, 0.850) and three post-cutoff Fujii estimates were added (0.804, 0.780, and 0.739). Validation maturity changed from 7/6/3/4 to 6/8/4/3 (external/internal hold-out/internal resampling/no independent validation) after Farrell was reclassified as same-cohort rather than external, the two Tavanaeian domain entries as internal hold-out rather than none, and Fujii as internal resampling. NR means no AUROC interval was reported or recoverable. Lu 2019 uses its reported interval (0.697–0.891); the Yang interval was derived from its reported standard error, and the Tavanaeian oral upper bound was capped at 1.00 and labelled.*

# **External Supplementary Files (provided separately)**

## **Audit workbook and analysis files (OSF repository)**

The current audit workbook and analysis kit contain the database strategies and aggregate yields, the 21-entry dataset, the 20-record plotted risk-of-bias set, the full-text exclusion log, the reconstruction-verification ledger, the 51-estimate AUROC inventory, the targeted top-up summary, and dependency-sensitivity inputs and outputs. The repository contains the submitted-version files; proof-stage corrections are supplied separately.

## **PRISMA-DTA and PRISMA 2020 checklists**

The completed PRISMA-DTA checklist maps reporting of the adapted, GRADE-informed certainty appraisal to item 24 of McInnes et al. (2018); item 24 is not presented as the appraisal method. Updated PRISMA-DTA and PRISMA 2020 checklists are included in the submission package.

## **PROSPERO protocol record**

The protocol was registered in the PROSPERO international prospective register of systematic reviews under registration number CRD420261370926. The full registration record (eligibility criteria, search strategy, planned analyses) is available at https://www.crd.york.ac.uk/prospero/.

## **Open Science Framework (OSF) repository**

The repository at https://osf.io/h358f/ (DOI: https://doi.org/10.17605/OSF.IO/H358F) contains publicly accessible submitted-version files. Proof-stage corrections are provided separately.

# **Reporting-guidelines compliance**

Reporting is guided by PRISMA 2020 and PRISMA-DTA; QUADAS-2 and PROBAST inform bias assessment, and an adapted, GRADE-informed appraisal rates certainty very low. The updated six-row/five-report Tier A/B core is primary and unpooled; the nine-row/seven-report bivariate result is the reference exploratory synthesis. Fujii is a post-cutoff targeted-top-up report. The five-row/four-report descriptive and eight-row/six-report bivariate subsets preserve the original cutoff as temporal robustness checks. Reviewer-level workflow limitations and repository-version status are stated in M7.
